# Transcript Profiling Identifies *Iqgap2^−/−^* Mouse as a Model for Advanced Human Hepatocellular Carcinoma

**DOI:** 10.1371/journal.pone.0071826

**Published:** 2013-08-12

**Authors:** Dmitri V. Gnatenko, Xiao Xu, Wei Zhu, Valentina A. Schmidt

**Affiliations:** 1 Department of Medicine, Stony Brook University, Stony Brook, New York, United States of America; 2 Department of Biomedical Engineering, Stony Brook University, Stony Brook, New York, United States of America; 3 Department of Psychiatry, Icahn School of Medicine at Mt Sinai, New York, New York, United States of America; 4 Department of Applied Mathematics and Statistics, Stony Brook University, Stony Brook, New York, United States of America; Institut für Pathologie, Germany

## Abstract

It is broadly accepted that genetically engineered animal models do not always recapitulate human pathobiology. Therefore identifying best-fit mouse models of human cancers that truly reflect the corresponding human disease is of vital importance in elucidating molecular mechanisms of tumorigenesis and developing preventive and therapeutic approaches. A new hepatocellular carcinoma (HCC) mouse model lacking a novel putative tumor suppressor IQGAP2 has been generated by our laboratory. The aim of this study was to obtain the molecular signature of *Iqgap2^−/−^* HCC tumors and establish the relevance of this model to human disease. Here we report a comprehensive transcriptome analysis of *Iqgap2^−/−^* livers and a cross-species comparison of human and *Iqgap2^−/−^* HCC tumors using Significance Analysis of Microarray (SAM) and unsupervised hierarchical clustering analysis. We identified the Wnt/β-catenin signaling pathway as the top canonical pathway dysregulated in *Iqgap2^−/−^* livers. We also demonstrated that *Iqgap2^−/−^* hepatic tumors shared genetic signatures with HCC tumors from patients with advanced disease as evidenced by a 78% mouse-to-human microarray data set concordance rate with 117 out of 151 identified ortholog genes having similar expression profiles across the two species. Collectively, these results indicate that the *Iqgap2* knockout mouse model closely recapitulates human HCC at the molecular level and supports its further application for the study of this disease.

## Introduction

Hepatocellular carcinoma or HCC, the most common primary malignancy of the liver, is one of the deadliest cancers worldwide, responsible for 700,000 deaths annually [1]. It remains a therapeutic challenge because the disease is mostly discovered in late stage when potentially curative surgical intervention is no longer an option. The 5-year survival rate in patients with advanced HCC is only 5%, and the mean survival of symptomatic patients is 2–3 months [2]. Finding novel therapeutic agents and biomarkers remains an urgent and yet often elusive task. HCC tumors feature high genomic heterogeneity due to the broad etiology of the disease, which includes chronic viral hepatitis B and C infections, cirrhosis, hepatic steatosis, and exposure to toxic agents such as aflatoxin B1 [3]. The heterogeneity and instability of human tumors pose a serious impediment to the identification of target genes for cancer therapy, making genetically well-defined mouse models increasingly important in cancer research.

Systematic transcriptome analysis of individual patient tumors and tumors from mouse models of cancer is an important tool for defining the intricate molecular networks of carcinogenesis. To this end, cross-species gene expression comparisons of animal models and human disease are a key step in validation of molecular mechanisms driving HCC pathogenesis [4]. Our previous work identified IQ-motif containing GTPase-activating protein 2 (IQGAP2) as a novel putative tumor suppressor in HCC [5]. IQGAP2 belongs to a family of highly homologous multidomain scaffolding proteins which also includes IQGAP1 and IQGAP3 [6]. The major function of these proteins consists of integration of Rho GTPase and Ca^2+^/calmodulin cellular signaling events with cytoskeletal dynamics [7]. Unlike the ubiquitously expressed and extensively studied IQGAP1 homolog, IQGAP2 is predominantly expressed in liver, and its physiological role remained poorly understood until a whole body *Iqgap2* knockout mouse was generated [5]. It was discovered that 86% of *Iqgap2^−/−^* mice developed liver tumors at between 18 and 24 months of age, whereas wild-type littermates were completely tumor-free at any age [5]. Both sexes were affected equally and no other malignancies were evident. *Iqgap2^−/−^* hepatic tumors displayed cellular alterations and histological features similar to that of human HCC [5]. The late onset of HCC development in *Iqgap2^−/−^* mice was consistent with the timeframe of human disease, which undergoes multiple phases and often takes decades to progress to carcinoma [8]. At the molecular level, there were indications of activation of the Wnt/β-catenin signaling pathway in *Iqgap2^−/−^* [5]. The relevance of the *Iqgap2^−/−^* mouse model to human disease was later suggested by finding that IQGAP2 protein expression was down-regulated in 78% of HCC patient tumors compared to normal liver tissue [9].

In this study, comprehensive age-dependent gene expression profiles of *Iqgap2^−/−^* livers were obtained using RNA microarray technology. We determined a transcript signature of HCC in the setting of IQGAP2-deficiency and confirmed deregulation of the Wnt/β-catenin signaling pathway as a major molecular event in HCC tumorigenesis in the absence of IQGAP2. We next demonstrated that hepatic tumors from the *Iqgap2^−/−^* mouse model accurately recapitulate many molecular signatures of human HCC using a HCC patient microarray data set and comparative functional genomics, confirming the *Iqgap2^−/−^* mouse as a novel translationally and clinically relevant animal model for HCC.

## Materials and Methods

### Mice

Generation of the *Iqgap2^−/−^* mice was previously described [5]. Both *Iqgap2^−/−^* and wild-type control mice were of 129 genetic background.

### Ethics Statement

All mice were handled according to guidelines for the humane care and use of experimental animals, and procedures were approved by the Stony Brook University Institutional Animal Care and Use Committee, protocol # 239805-5.

### Microarray analysis

Livers were extracted from 6-month-old and 24-month-old male *Iqgap2^−/−^* (KO) and sex- and age-matched wild-type (WT) mice (N  =  3 per group), and total RNA was isolated from fresh tissue using the Trizol method (Invitrogen, Carlsbad, CA). For the 24-month-old KO group, total RNA was extracted from liver tumors; for all other groups normal liver tissue was used as a source of total RNA. RNA quality and yield were assessed by Bioanalyzer 2100 (Agilent, Santa Clara, CA).

Relative gene expression was evaluated using the GeneChip® Mouse Genome 430 2.0 Array (Affymetrix, Santa Clara, CA). This single-color array represents approximately 14,000 well-characterized mouse genes. Hybridization was performed according to the manufacturer's protocol. Briefly, 5 µg of total RNA was used for reverse transcription reaction with a SuperScript II reverse transcription kit (Invitrogen). cDNA was purified by phenol/chloroform extraction followed by ethanol precipitation. *In-vitro* transcription (IVT) was performed using an IVT kit (Affymetrix). Products of the IVT reaction were purified using the RNeasy kit (Qiagen, Germantown, MD) with DNAse treatment. Twenty µg of cRNA were fragmented using Affymetrix 5X fragmentation buffer and 15 µg of fragmented cRNA was used for hybridization. Hybridization cocktail was incubated at 95°C for 5 minutes, followed by 5 minutes at 45°C, centrifuged at 18,000 g at room temperature for 5 minutes and applied to Affymetrix MG 430 2.0 arrays. Hybridization was conducted overnight at 45°C. Arrays were washed and stained according to Affymetrix protocols, then scanned using an Affymetrix 7G scanner and pre-processed using GeneChip Operation System v.1.4 software to generate CEL files. Minimum Information about Microarray Experiment (MIAME)-compliant data was deposited to the Gene Expression Omnibus (GEO) public database at NCBI (http://www.ncbi.nlm.nih.gov/geo/) and are accessible through GEO Accession numbers GPL1261 (platform) and GSE46646 (samples).

### Gene expression profiling of *Iqgap2^−/−^* mouse model and unsupervised hierarchical clustering analysis

A comparative functional genomics approach based on methods previously described [10], [11] was adapted in our study. Microarray data were imported into the R Bioconductor Simpleaffy package (http://www.bioconductor.org). The Robust Multi-Array Average (RMA) procedure was used to normalize the data [11] and log2 transformation was applied using the default settings of the RMA protocol. To identify differentially-expressed transcripts, Significance Analysis of Microarray (SAM) [12] was applied with a fold change cutoff of 3 and the false discovery rate (FDR) controlled at no more than 0.05. SAM analysis was performed to identify differentially expressed genes between each two pairs of the four experimental and control groups (KO 6-month-old, KO 24-month-old, WT 6-month-old and WT 24-month-old). The union of all differentially expressed genes was pooled across the 4 comparisons and used to identify sub-patterns of the selected gene profiles. Specifically, an unsupervised hierarchical clustering analysis was performed to categorize genes into different clusters based on a distance metric (1-Pearson correlation) and an average linkage distance between clusters. All data were standardized to a mean of 0 and a standard deviation of 1 prior to the clustering procedure and only the 4 group means were used in the analysis. A total of 11 clusters were identified using a consensus of 3 popular cluster number estimation indices, including the weighted inter- to intra- cluster ratio, homogeneity separation [13] and the Silhouette index [14], all implemented in Matlab (R2009b, Mathworks®, Natick, MA). Each of the 11 clusters was characterized by its group mean patterns. Functional signaling pathway and network analysis was subsequently performed using the Ingenuity Pathway Analysis (IPA) tool (Ingenuity®, Redwood City, CA).

### Quantitative real-time PCR analysis (Q-PCR)

Mouse Wnt Signaling Pathway RT^2^ Profiler PCR Array (Qiagen) was used for RNA transcript profiling. This PCR array allows simultaneous profiling of 84 genes and includes control housekeeping genes for data normalization. Five ug of RNA samples from 24-month-old KO and WT mice used for microarray analysis were subjected to reverse transcription using the SuperScript II reverse transcription kit. Resulting cDNA was diluted 1∶15 and PCR amplified using a DNA Engine Opticon 2 thermocycler (Bio-Rad, Hercules, CA) and cycling conditions recommended by Qiagen. Opticon Monitor 3 v.1 software (Bio-Rad) was used for initial data extraction. Data analysis was performed using the RT^2^ Profiler PCR Array Data Analysis Template v3.3 from Qiagen. A fold change of 3, p-value <0.05 was used as a cutoff. Data were normalized to five housekeeping genes – *Gusb, Hprt1, Hsp90ab1, Gapdh* and *Actb*.

### Comparison of HCC gene expression profiles between mouse and human tumors

To compare HCC gene expression profiles of *Iqgap2^−/−^* mouse model and patient tumors, SAM and unsupervised hierarchical clustering statistical analyses were performed on a human HCC microarray data set GSE6222 [15] downloaded from the GEO database at http://www.ncbi.nlm.nih.gov/gds/. The SAM analysis was modified by altering the fold change threshold cutoff from 3 to 2, FDR≤0.05, due to an insufficient number of differentially expressed overlapping ortholog transcripts detected between the *Iqgap2^−/−^* mouse microarray data set, consisting of 6 samples, (3 normal liver samples from 24-month-old WT mice and 3 HCC tumors from 24-month-old KO mice) and the human array data set using SAM with a fold change cutoff ≥3. RMA normalization and SAM analysis were performed using the same settings (fold change ≥2, FDR≤0.05). Expression profiles of genes from the *Iqgap2^−/−^* mouse and the human HCC microarray data sets were normalized to a mean of 0 and standard deviation of 1 to make the scale of gene expression profiles comparable.

A subsequent comparative functional genomics procedure [11] was performed to map mouse HCC gene expression profiles to their human counterparts. An initial genetic orthologs search was carried out with a custom-developed R script by referencing the orthologs/homologs annotation file (http://www.affymetrix.com/support) of Affymetrix Mouse 430 2.0 array. Affymetrix probe ID sets from both mouse and human platforms were first converted to common orthologous entries represented by gene symbols before further cross-platform validation. Affymetrix probe sets not sharing a common orthologous entry (gene symbol) between the mouse and its corresponding human HCC profiles were excluded. Often, the Affymetrix probe sets and the corresponding orthologous genes have more complex relations than a one-to-one match. In the event one-to-multiple corresponding links between an ortholog gene symbol and an Affymetrix probe ID were observed, the algorithm used the average of the duplicates and compressed the multiple entries to a single term for subsequent calculations. Conversely, in the event one Affymetrix probe ID corresponded to multiple ortholog genes, the expression value of the probe ID was split and assigned to their orthologous entries evenly. This ensured that the intricate multiple-to-multiple relationship between the Affymetrix probe IDs and ortholog genes is reduced to a set of simple average and split calculations. This process was performed repeatedly for each of the Affymetrix probe IDs.

After the Affymetrix probe IDs conversion, mouse and human datasets were integrated into one dataset and a hierarchical clustering analysis was performed to examine the similarity between the mouse and the human HCC expression profiles. The unsupervised clustering procedure was conducted in an array dimension based on 1- Pearson correlation distance and using the average linkage method to compute the distance between clusters, where the distance between two clusters is the average distance of all point pairs from different clusters [16]. All data sets were scaled to the range from −3 to 3. A heat map was then generated to present the clustering results.

## Results

### Microarray analysis of HCC development in *Iqgap2^−/−^* mice

**Figure 1:**
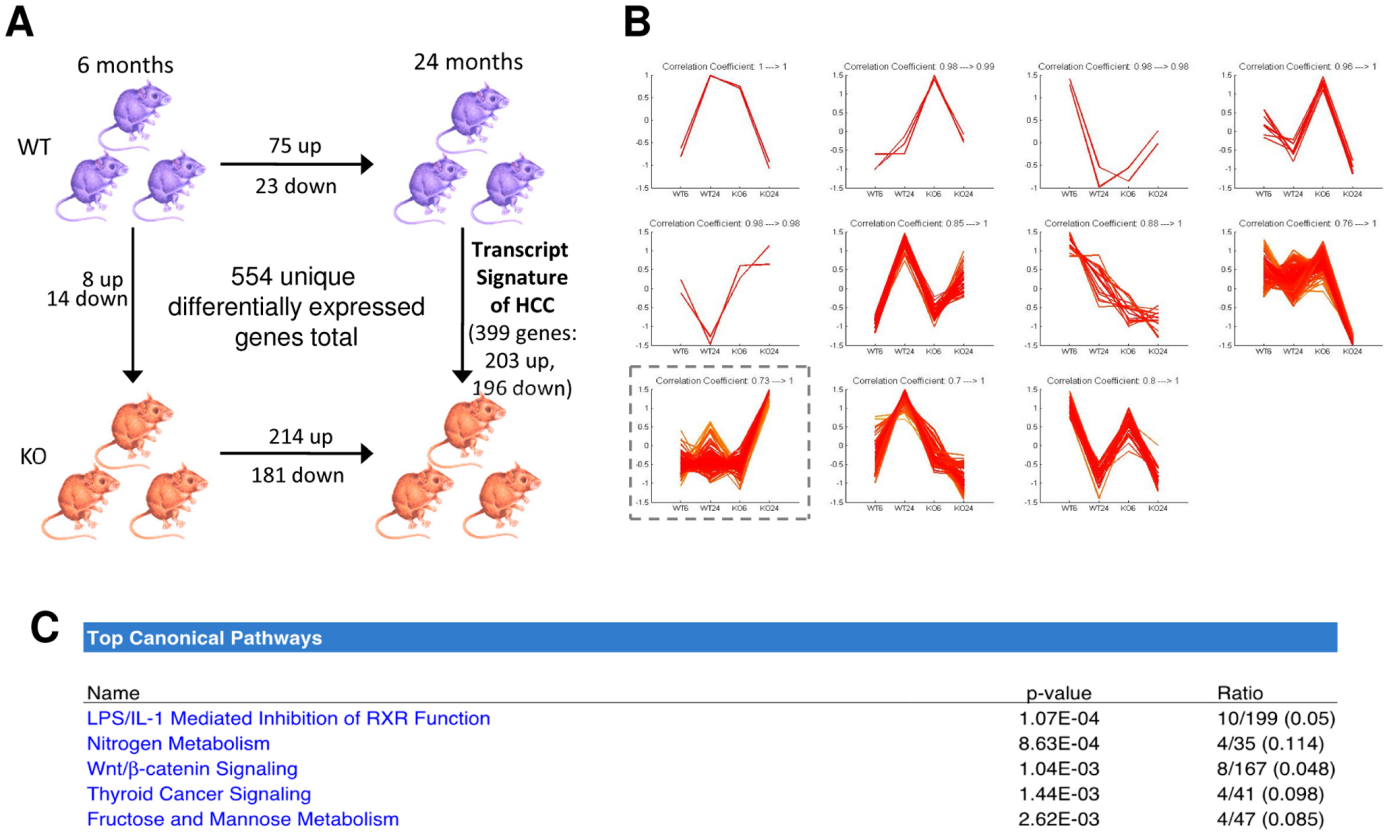
Hepatic RNA microarray analysis of *Iqgap2^−/−^* mice. **A**: Schema of a four-way microarray analysis of *Iqgap2^−/−^* knockout (KO) and wild-type (WT) mouse livers at 6 months and 24 months of age. Four-way comparison using SAM identified 554 genes that were differentially expressed among four groups (cutoff fold change ≥3 and FDR≤0.05). **B**: Unsupervised hierarchical cluster analysis identified 11 subsets of genes (clusters) within 554 genes that change expression in similar pattern across four groups; **C**: Top canonical biological pathways for genes from the cluster # 9 as identified by Ingenuity Pathway Analysis (IPA).

Transcript profiles of four groups of mouse livers (N  =  3 in each group) were compared using Affymetrix microarray technology. The groups included livers from 6- and 24-month-old wild-type (WT) mice and 6- and 24-month-old (KO) *Iqgap2^−/−^* mice. SAM algorithm was used for initial four-way comparison to identify differentially expressed genes among four groups with a cutoff fold change ≥3 and FDR≤0.05. A total of 554 genes were shown to be differentially expressed among these four groups (**Fig. 1 A**). Unsupervised hierarchical clustering analysis was performed to identify genes that behave in a similar pattern across four groups (**Fig. 1 B**). All differentially expressed genes were grouped in 11 clusters based on a pattern of expression change. Within each cluster, individual genes changed their expression levels between the groups in a similar manner. The resulting clusters were highly unbalanced - four clusters had only 3 genes or less (clusters # 1–3 and 5), whereas clusters # 8 and 9 had 141 and 247 genes, respectively. The largest cluster, cluster # 9 (marked with a dashed line), included genes that are significantly overexpressed in KO HCC tumors compared to all three non-tumor sample groups (WT of both ages and the 6-month-old KO). To identify biological pathways associated with the identified differentially expressed genes and to examine the enrichment of molecular pathways for each gene cluster, IPA platform was used next. IPA is a large curated database of molecular interactions, gene-to-phenotype associations, and chemical knowledge for biological pathway analysis. IPA examination of gene cluster # 9 identified the Wnt/β-catenin signaling pathway among the top canonical pathways relevant to the development of HCC in the *Iqgap2^−/−^* model (**Fig. 1 C**). The canonical Wnt/β-catenin pathway has been shown to be activated in 33–67% of HCC patient cases studied [17]. The nitrogen metabolism pathway, the number two pathway identified as dysregulated in the *Iqgap2^−/−^* HCC tumor gene cluster #9, is closely associated with the Wnt/β-catenin pathway in liver [18]. Finally, the top pathway found to be dysregulated in gene cluster #9 using IPA was the LPS/IL1 Mediated Inhibition of RXR Function pathway, known to be associated in liver with chronic inflammation and injury caused by various toxic agents and carcinogens [19], [20]. This pathway is also involved in regulating lipid metabolism. The latter is especially relevant for the *Iqgap2^−/−^* model, since these mice also display a metabolic phenotype linked to a defect in hepatic lipogenesis and fatty acid uptake [21].

### Analysis of genes potentially involved in initiation of HCC development

Our microarray data demonstrate that in WT livers, 98 genes changed expression with age: 75 were upregulated and 23 - downregulated (**Fig. 1 A**). Significantly more genes changed their expression with age in KO livers – 395 genes total. Of those, 214 genes were upregulated and 181 were downregulated at 24 months compared to 6 months of age (**Fig. 1 A**). Many of these genes may play a role not in normal liver ageing but in HCC triggering and development. To narrow down the search of genes – potential drivers of HCC development in the absence of IQGAP2, we focused on genes that change expression with age in KO, but not in WT livers. Using SAM algorithm (fold change ≥3, FDR≤0.05), 371 genes were identified as differentially expressed between the age of 6 and 24 months in KO livers only (see a list of genes in **Table S1**). Among the genes that most significantly increased expression from 6 to 24 months in KO but not WT livers were α–fetoprotein, AFP (*Afp*, 17-fold), Nemo-like kinase (*Nlk*, 11-fold), trans-acting transcription factor 5 (*Sp5*, 11-fold) and Wnt-inhibitory factor 1 (*Wif1*, 9-fold). AFP is overexpressed in human primary liver cancers and has been used broadly as a biomarker for HCC [22]. Both Wnt-inhibitory factor and Nemo-like kinase belong to the Wnt/β-catenin signaling pathway [23]. Targeted disruption of Nemo-like kinase inhibits tumor cell growth by simultaneous suppression of cyclin D1 and cyclin-directed kinases in human HCC [24]. Elevated expression of Sp5 was noted in several human cancers including HCC, gastric cancer, and colon cancer [25], and it is considered a therapeutic target for HCC [26]. Genes that were down-regulated in KO livers with age included insulin-like growth factor binding proteins 2, 3 and 5 (*Igfbp2, Igfbp3* and *Igfbp5*, a fold change of 21-, 10- and 3-fold, respectively) and cadherin1 (*Cdh1*, 11-fold). Reduced expression of these proteins has been previously characterized in human HCC [27]–[29]. Taken together, these results suggested significant similarities in transcriptome between the *Iqgap2^−/−^* mouse model and human HCC.

To gain further insight into molecular mechanisms regulating HCC development in IQGAP2-deficiency, the IPA database was utilized for subsequent pathways network analysis. Analysis of a subset of 371 genes that were differentially expressed with age (i.e. between 6-month-old and 24-month-old groups) in KO but not in WT livers confirmed that the Wnt/β-catenin signaling pathway is the top canonical pathway altered in HCC tumors in the *Iqgap2^−/−^* mouse model (**Table 1 A**). Eight genes in the 371 gene subset (*Axin2*, *Ccnd1*, *CD44*, *Nlk*, *Sfrp2*, *Tcf7*, *Ubd* and *Wif1*) are implicated in Wnt/β-catenin signaling. Interestingly, all these genes were found in gene cluster # 9 (**Fig. 1 B**), suggesting that they change expression concordantly during HCC development. Furthermore, IPA analysis of network functions of the 371 gene subset demonstrated that several networks, including cancer networks and lipid metabolism network, are associated with HCC development in *Iqgap2^−/−^* mice (**Table 1 B**). Alterations in cellular lipid metabolism resulting in obesity, diabetes and non-alcoholic fatty liver disease (NAFLD) are a well-established risk factor for HCC [30], [31]. Finally, HCC was ranked the second highest in the IPA-Tox (an IPA tool that matches gene expression changes to biological mechanisms related to toxicology) network functions (**Table 1 C**), with 9 genes overexpressed in *Iqgap2^−/−^* liver tumors directly linked to human HCC (*Afp*, *Ect2*, *Icam1*, *Nqo1*, *Pdgfc*, *Pnpla3*, *Socs3*, *Tuba8* and *Ube2c*), thus verifying the identity of *Iqgap2^−/−^* hepatic tumors. Of note, liver steatosis was ranked the highest among IPA-Tox functions, suggesting serious abnormalities in fatty acid metabolism in *Iqgap2^−/−^* livers. Seven genes overexpressed in *Iqgap2^−/−^* HCC tumors were related to hepatic steatosis – *Acsl4*, *Ccnd1*, *CD44*, *Fabp5*, *Lpl*, *Pdgfc* and *Pparg*. Again, this is in agreement with reported earlier aberrations in fatty acid uptake and lipogenesis in *Iqgap2^−/−^* livers [21].

**Table 1:** Ingenuity Pathway Analysis of biological functions of 371 genes that change expression with age in *Iqgap2^−/−^* but not in WT livers, leading to the development of HCC.

A. Top Canonic Pathways	
**Pathway**	**p-value**
1. Wnt/β-catenin signaling	1.64×10^−4^
2. Role of macrophages, fibroblasts and endothelial cells in rheumatoid arthritis	2.54×10^−4^
3. LPS/IL-1 mediated inhibition of RXR function	5.14×10^−4^
4. Thyroid cancer signaling	7.03×10^−4^
5. Atherosclerosis signaling	2.51×10^−3^
**B. Top Associated Network Functions**	
**Network**	**Score**
1. Cancer, Genetic Disorder, Reproductive System Disease	46
2. Cancer, Genetic Disorder, Respiratory Disease	42
3. Lipid Metabolism, Molecular Transport, Small Molecule Biochemistry	31
4. Developmental Disorder, Gastrointestinal Disease, Hepatic System Disease	24
5. Lipid Metabolism, Molecular Transport	24
**C. Top Tox Functions, Hepatotoxicity**	
**Disease**	**p-value**
1. Liver steatosis	2.09×10^−4^
2. Hepatocellular carcinoma	2.81×10^−4^
3. Glutathione depletion in liver	1.58×10^−2^
4. Liver cirrhosis	1.83×10^−2^
5. Liver dysfunction	1.83×10^−2^

The top five canonic biological pathways (**A**), top five associated networks (**B**) and top five Tox functions (**C**) are shown.

### Q-PCR validation of the Wnt/β-catenin signaling activation in *Iqgap2^−/−^* livers

**Figure 2:**
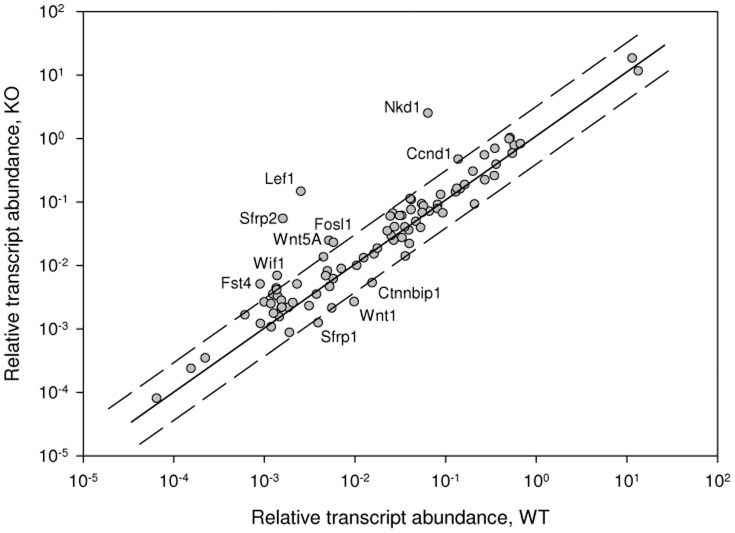
Q-PCR validation of the Wnt/β-catenin signaling pathway alterations in *Iqgap2^−/−^* livers. The same liver RNA samples used for microarray (24-month-old WT, N  =  3, and 24-month-old KO, N  =  3) were analyzed using the Mouse Wnt Signaling Pathway RT^2^ Profiler PCR Array. 11 genes belonging to the pathway and found to be differentially expressed in *Iqgap2^−/−^* livers are shown.

The Mouse Wnt Signaling Pathway RT^2^ Profiler PCR Array (Qiagen) was used to validate the microarray data and confirm the activation of the canonical Wnt/β-catenin pathway in *Iqgap2^−/−^* hepatic tumors. The same liver RNA samples used for microarray (24-month-old WT, N  =  3, and 24-month-old KO, N  =  3) were analyzed by qRT-PCR. Since no differences in the Wnt/β-catenin pathway status were detected between the genotypes in the samples from 6-month-old mice by microarray, qRT-PCR validation was limited to the samples from the 24-month-old group. As a result, 8 genes related to the Wnt/β-catenin signaling pathway (*Lef1*, *Nkd1*, *Ccnd1*, *Sfrp2*, *Fosl1*, *Wnt5A*, *Wif1* and *Fgf4*) were identified as up-regulated in KO tumors compared to 24-month-old WT tumor-free livers, while another 3 genes from the pathway (*Ctnnbip1*, *Wnt2* and Sfrp1) were down-regulated in KO tumors (**Fig. 2**). Notably, overexpression of the *Ccnd1* gene encoding cyclin D1, one of the main nuclear targets of β-catenin, has been consistently detected in this study using all approaches and correlated with cyclin D1 protein overexpression observed previously in *Iqgap2^−/−^* livers [5]. These results confirmed our microarray - based IPA finding on the important role of Wnt/β-catenin signaling in HCC development in *Iqgap2^−/−^* mice.

### RNA transcript signature of HCC in *Iqgap2^−/−^* mouse model

To more comprehensively examine molecular changes responsible for the *Iqgap2^−/−^* tumorigenic phenotype and to gain an insight into mechanisms underlying the tumor suppressive function of IQGAP2, we next compared age-dependent changes in transcript expression between WT and KO livers. The objective was to identify genes and pathways that may trigger the development of HCC in the absence of IQGAP2. Using SAM analysis (fold change ≥3, FDR≤0.05), we found that at the age of 6 months, the difference in hepatic transcript expression between KO and WT mice was minimal. Only 8 genes were up-regulated and 14 genes down-regulated in KO livers compared to WT in this age group. At the age of 24 months, however, a considerably larger number of genes showed significant differential expression between WT (tumor-free) and KO (HCC) livers - 399 genes total, with 203 up-regulated and 196 down-regulated in HCC compared to normal WT liver (**Fig. 1 A** and **Table S2**). This subset of genes represents a “transcript signature” of HCC in the *Iqgap2^−/−^* mouse model. It included many genes known to play an important role in human HCC. Thus, the following genes had elevated levels in KO HCC tumors compared to WT livers: platelet derived growth factor alpha (*Pdgfa*, 5-fold), cyclin D1 (*Ccnd1*, 3-fold), cyclin B2 (*Ccnd2*, 5-fold), fibroblast growth factor 21 (*Fgf21*, 9-fold) and E2F transcription factor 7 (*E2f7*, - 4-fold). Overexpression of hepatocyte-specific *Fgf21* has been demonstrated in human HCC [32]. This protein also plays a key role in hepatic fatty acid metabolism in mice [33]. Three genes encoding insulin-like growth factor binding proteins were significantly downregulated in KO HCC tumors (*Igfbp2*, 14-fold, *Igfbp3*, 6-fold, and *Igfbp5*, 4-fold). An important role of insulin-like growth factors, their receptors and binding proteins has been shown in early hepatic carcinogenesis [34]. In four independent transgenic mouse models, hepatocyte-specific expression of insulin-like growth factor II (IGFII) induced tumorigenesis associated with HCC development [35]. Interestingly, *Igfbp1* was overexpressed in KO livers at 6 months of age, when KO livers still exhibited normal morphology and histology, but was not identified as differentially expressed at the age of 24 months in KO liver tumors. Overexpression of IGFBP-1 is linked to deregulation of metabolism of insulin-like growth factors (ILGFs) during HCC development [36].

**Figure 3:**
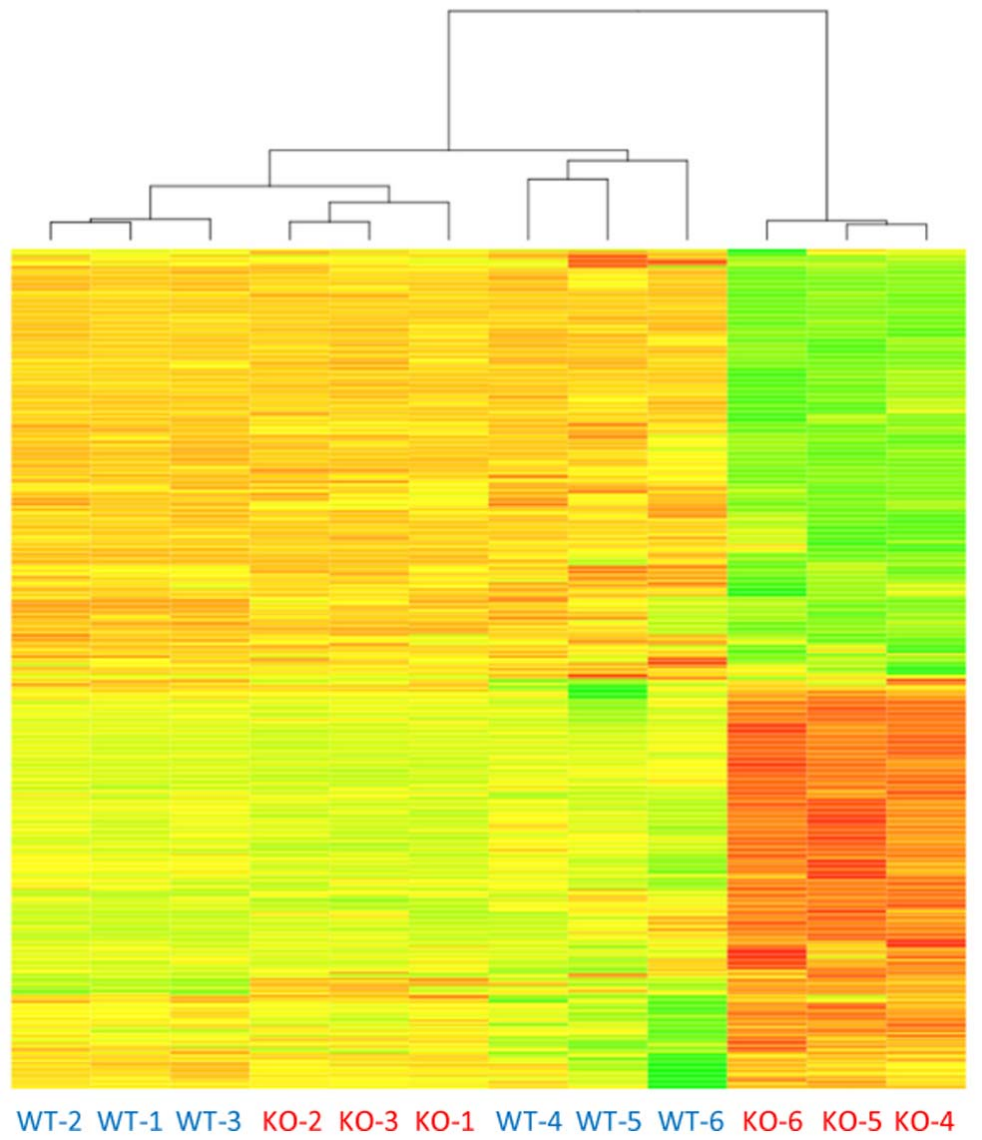
Unsupervised hierarchical clustering analysis of the 399 genes subset representing the *Iqgap2^−/−^* HCC transcript signature. The two-dimension hierarchical clustering procedure was performed based on the 1- Pearson correlation distance and the average linkage method. All data were centered by rows to mean 0 and standard deviation of 1, meanwhile the data range was confined to −3 to 3 for a more comparable scale. The data are presented in a matrix format with columns representing individual samples and rows representing genes, thereby each cell in the matrix represents the expression level of a gene feature in an individual sample. The red and green colors in cells reflect high and low expression levels, respectively, as indicated in the fold-change scale bar. Tumor-free samples have blue font labels and HCC samples have maroon font labels.WT-1, WT-2, WT-3 – wild-type liver samples from 6-month-old mice; KO-1, KO-2, KO-3 - *Iqgap2^−/−^* liver samples from 6-month-old mice; WT-4, WT-5, WT-6 - wild-type liver samples from 24-month-old mice; and KO-4, KO-5, KO-6 - *Iqgap2^−/−^* liver tumor samples from 24-month-old mice. Note that the three *Iqgap2^−/−^* HCC tumor samples from 24-month-old mice show a distinct pattern and form a separate cluster. The rest of the samples have more similar transcript profiles with the highest similarity found between livers from the younger (6-month-old) mice irrespectively of genotype.

A heat diagram (**Fig. 3**) shows that nearly half of the genes in the 399 gene subset were up-regulated in the 24-month-old KO HCC tumors (203 up-regulated genes vs. 196 down-regulated). HCC tumors from all three 24-months-old KO mice formed a distinct cluster, implying that gene expression patterns of KO HCCs are clearly homogeneous. A striking difference in color indicates that the KO HCC group is significantly different from the three other groups of non-tumor samples. Unsupervised hierarchical clustering analysis confirmed this observation. WT and KO samples were grouped in separate clusters according to age, with no exceptions. Three sets of non-tumor samples (6-month-old WT, 6-month-old KO and 24-month-old WT) were grouped together in one large cluster, whereas the 24-month-old KO group was clustered separately. Inter-group distances separating the 24-month-old KO group from all other groups were significantly greater compared to the distances between the other groups. Inter-sample distances within a group were the largest in the 24-month-old WT group, presumably due to increasing gene expression variability with age. In contrast, inter-sample distances within the 24-month-old KO group were surprisingly small, indicating very consistent gene expression in KO HCC tumors.

### *Iqgap2^−/−^* mouse model recapitulates molecular features of advanced human HCC

To establish relevance of the *Iqgap2^−/−^* mouse model to human disease, expression profiles of *Iqgap2^−/−^* HCC tumors were compared to a human HCC microarray data set, GSE6222. The GSE6222 data set [15] contains 4 different patient primary HCC tumors (early stage T1), 6 unrelated patient intrahepatic metastatic HCC tumors (late stage T3) and 2 normal liver samples. This human data set was selected from the GEO database based on its array platform (Affymetrix Human Genome U-133-plus2), which has similar to the GeneChip® Mouse Genome 430 2.0 Array probe set system, aiding cross-platform orthologous mapping. Another advantage of the GSE6222 data set was that it offers specific diagnostic delineations of HCC progression providing comparative expression profiles of stage T1 and T3 tumors. The gene features selected as a result of cross-validation between mouse and human arrays were initially obtained from SAM analysis with a fold change threshold of 2 and FDR≤0.05, allowing the inter-species mapping using only significant transcript features relevant to HCC. The subsequent data integration based on the common orthologous entries further eliminated genes that were not mutually present on both species arrays. As a result, the above sequential filtering procedures reduced the shared orthologous genes between the mouse KO HCC data set and the human GSE6222 data set to 151 common genes (**Table S3**).

**Figure 4:**
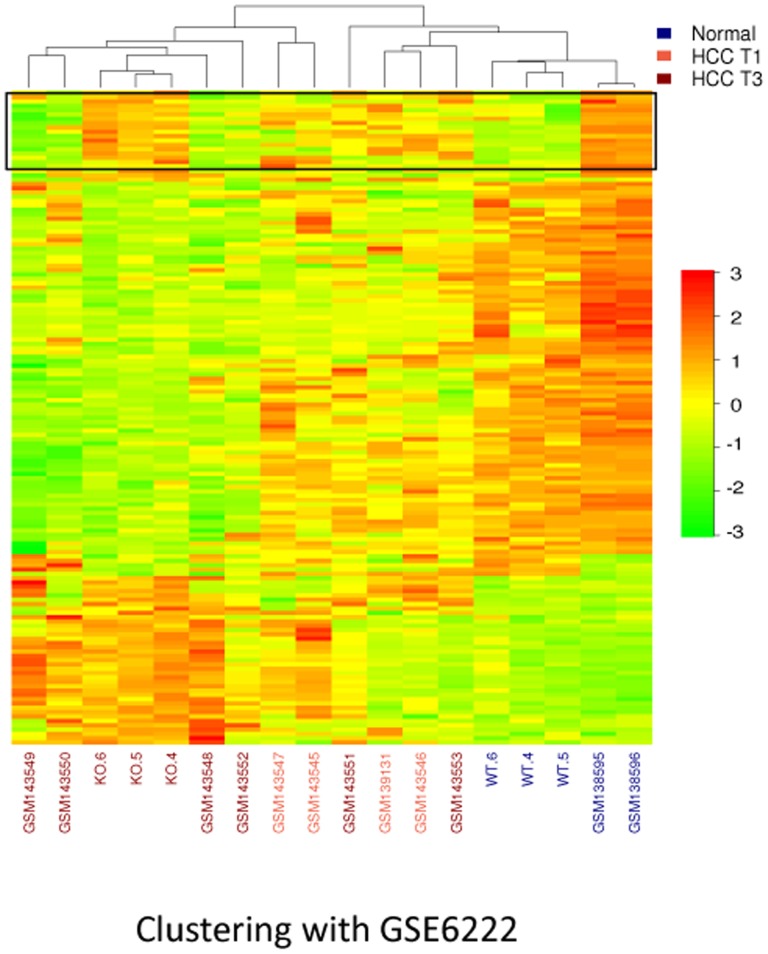
Comparison of HCC transcript profiles of *Iqgap2^−/−^* mouse model and human HCC. Cross-species clustering profiles were obtained using comparative functional genomics approach. The clustering dendrogram was generated based on 1 - Pearson correlation and an average distance linkage. The data scale was also confined to a range of −3 to 3 for a more comparable heat map. Hierarchical clustering analysis was performed on 151 ortholog genes shared between 24-month-old wild-type and the age-matched *Iqgap2^−/−^* mice (N = 3 in each group, see **Figure 3** legend), and the human GSE6222 integrated microarray data set. This data set included four T1 HCC tumors (early stage HCC), six T3 HCC tumors (advanced HCC), and 2 normal liver controls. Mouse and human tumor-free samples have blue font labels; human T1 HCC samples have red font labels; and *Iqgap2^−/−^* HCC and human T3 HCC samples have maroon font labels. All three *Iqgap2^−/−^* HCC samples (KO.4 through KO.6) co-clustered with four out of six human T3 HCC samples. Genes differentially expressed between mouse and human livers are enclosed in a box at the top of the heat diagram.

The comparative functional genomics evaluation using the hierarchical clustering method showed a clear delineation between normal livers and HCC tumors across two species (**Fig. 4**). In the KO HCC vs. human GSE6222 data set, the mouse samples generally fell into the same super-cluster with the human HCC yet retained a certain level of independence due to differential expression of a set of 24 mouse genes (framed in **Fig. 4**). The mouse-to-human concordance rate, defined by fold change, was exceptionally high (78%), with 117 out of 151 orthologs having similar expression profile across the two species. Additionally, since the GSE6222 dataset contained more specific phenotypic levels of HCC progression, it enabled comparison of the *Iqgap2^−/−^* HCC to the gene expression profiles of human HCC sub-types. A hierarchical clustering heat map (**Fig. 4**) indicates that the transcript signature of all three KO HCC tumors resembled the advanced (T3) HCC in humans. Taken together, the functional genomics analysis of *Iqgap2^−/−^* HCC tumors and human HCC data sets has shown a high level of consistency of transcript signatures across species and platforms. The hierarchical clustering results from this analysis indicated a strong predictive power of expression profiles of the identified mouse genes for human disease.

## Discussion

Overall survival of patients with HCC has not improved in the last two decades and late-stage HCC still has an extremely unfavorable prognosis. Development of much needed new targeted therapies and prognostic biomarkers necessitates the availability of well-characterized mouse models of HCC closely reproducing the molecular pathogenesis of the human disease. Genetically engineered mouse models are advantageous over chemically induced models because they allow us to closely follow and ascertain specific physiologically relevant molecular pathways and their contribution to HCC development. Hepatocarcinogenic agents used in chemically induced rodent models may be metabolized through distinct metabolic pathways in mouse vs. humans, making data extrapolation to humans difficult. While a number of genetically engineered HCC mouse models have been generated over the years, the majority are transgenic models overexpressing either well-characterized oncogenes and growth factors (e.g. *Myc* and *Tgfa*) or HBV genomic fragments and HCV core proteins (reviewed in [37]). One of the best characterized among a few available knockout mouse models of HCC is *Mdr2^−/−^* mouse, lacking a biliary transporter protein [38]. Our conventional knockout *Iqgap2^−/−^* mice represent a new HCC model involving a tumor suppressor not studied before. Furthermore, HCC mouse models involving animals of 129 genetic background, the background of the *Iqgap2^−/−^* mice, are significantly underrepresented [10]. It is important because different genetic backgrounds may convey distinct susceptibility to hepatocarcinogenesis.

In this study, we applied comparative functional genomics to evaluate the global expression signatures of orthologous genes between human HCC tumors and liver tumors derived from the IQGAP2-deficient mouse model. Spontaneous development of HCC in the majority of *Iqgap2^−/−^* mice suggests a protective role of IQGAP2 protein in liver, identifying it as a novel tumor suppressor. While tumors were detected through gross examination of excised livers in mice of 18 months of age and older, an elevated serum level of aspartate aminotransferase (AST) was detected in *Iqgap2^−/−^* mice as early as 4 months of age [5]. This suggests that early onset of HCC in the form of dysplastic and neoplastic hepatic lesions might be evident in younger *Iqgap2^−/−^* mice. Initial probing into the mechanism behind the potential tumor suppressive function of IQGAP2 in liver revealed that *Iqgap2^−/−^* HCC tumors were characterized by an 8-fold increase in cyclin D1 protein levels (a β-catenin nuclear target), β-catenin translocation from the cellular membrane, accumulation of its dephosphorylated (active) form and loss of membrane E-cadherin expression [5]. These findings linked HCC development in *Iqgap2^−/−^* mice to the canonical Wnt/β-catenin pathway activation.

Here we report that the Wnt/β-catenin signaling pathway is among the top three canonical signaling pathways dysregulated in *Iqgap2^−/−^* hepatic tumors. Many transcriptional markers of the Wnt/β-catenin pathway activation, including cyclin D1, were up-regulated in *Iqgap2^−/−^* liver tumors. Our findings are in agreement with a recent study involving whole-exome sequencing of 24 patient HCC tumors confirming the Wnt/β-catenin pathway as the most altered signaling pathway in HCC [39]. β-catenin is one of the crucial downstream effectors of the extensively studied canonical Wnt signaling pathway associated with HCC and regulating cell cycle, cell growth, and proliferation [40]. Mutations in the β-catenin gene are considered to be an early event in hepatocarcinogenesis [41]. Also, β-catenin activating mutations seem to be particularly common in HCCs associated with chronic HCV infection [42]. Recently, deregulation of the Wnt/β-catenin pathway has been used to identify two distinct molecular subclasses of human HCC linked to different prognosis [43]. The CTNNB1 subclass was characterized by activating mutations in hepatic *CTNNB1* gene, up-regulation of Wnt nuclear targets and overall a less aggressive disease. Conversely, HCC tumors from the Wnt-TGFβ subclass had no mutations in β-catenin encoding gene, absent nuclear β-catenin and a more aggressive phenotype [43], [44]. Our previous studies showed that *Iqgap2^−/−^* hepatic tumors displayed β-catenin mutations and intense cytoplasmic and nuclear staining [5], therefore categorizing *Iqgap2^−/−^* HCC as belonging to the CTNNB1 subclass. In this respect, the *Iqgap2^−/−^* model shows similarities with *Myc* and *Myc/E2f1* transgenic mouse models [11].

β-catenin activation alone, however, does not seem to cause progression to HCC from a nonmalignant state. Studies with β-catenin transgenic mouse models indicate that dysregulated Wnt/β-catenin signaling can cause severe hepatomegaly, but is not sufficient for carcinogenic transformation [42]. This suggests that IQGAP2, being a scaffolding protein, may realize its tumor suppressing function through cross-linking several signaling pathways in liver, and that the Wnt/β-catenin pathway is one of them.

We next used our *Iqgap2^−/−^* model to identify potential molecular drivers of HCC initiation and development. This was accomplished by identifying 371 genes that changed expression between the age of 6 and 24 months in *Iqgap2^−/−^* livers only and not in WT livers. Among these, a short list of potential cancer molecular drivers highly relevant for HCC, whose expression was most significantly altered in *Iqgap2^−/−^* livers in age-dependent manner, included *Afp*, *Nlk*, *Sp5*, and *Wif1* (up-regulated expression); and *Igfbp3*, *Igfbp5*, and *Cadherin1* (down-regulated expression). Furthermore, among the 371 genes, a group of 9 genes changed its expression with age in the opposite direction in KO vs. WT livers. It included *Inhbe* and *Neat1* (upregulated in 24-month-old KO vs. 24-month-old WT), and *Ifi202b*, *Gvin1*, *Fabp7*, *Cyp17a1*, *Rasgef1b*, *Gm10567* and *Cyp4a14* (downregulated in 24-month-old KO vs. 24-month-old WT). The significance of such differential expression merits further investigation.

A transcript signature of HCC tumors in the context of IQGAP2-deficiency was also established in this study. This was accomplished by comparing gene expression changes in WT and *Iqgap2^−/−^* livers between the ages 6 and 24 months. While at the age of 6 months the difference in hepatic gene expression between WT and *Iqgap2^−/−^* was minimal and involved only 22 genes, by the age of 24 months, however, 399 genes showed differential expression in *Iqgap2^−/−^* livers compared to WT. These 399 genes constitute a transcript signature of *Iqgap2^−/−^* HCC. Among the genes with the most significantly altered expression in *Iqgap2^−/−^* HCC tumors were *Pdfa5*, *Ccnd1*, *Ccnd2*, *Fgf21*, and *E2f7* (up-regulation); and *Igfbp2*, *Igfbp3*, and *Igfbp5* (down-regulation). Of note, an analysis of mRNA expression profiles of adjacent non-tumorous liver tissue from 24-month-old KO mice may provide additional insights into the molecular mechanisms of liver cancer pathogenesis in our mouse model and will be conducted in the future. A comparison of expression profiles of HCC tumors and corresponding adjacent tissue from the same KO mouse will produce a list of individual genes associated with the development of HCC, whereas the analysis reported here identifies the general transcriptomic differences between WT and KO livers, the differences that presumably predispose KO mice to the development of HCC.

Finally, comparative functional genomics tools were used to perform a mouse-to-human comparison of HCC transcript signatures to test the relevance of the *Iqgap2^−/−^* mouse model to human HCC. We found remarkable consistency between gene expression profiles of *Iqgap2^−/−^* hepatic tumors and human HCC tumors, late-stage HCC in particular, reflected in a mouse-to-human concordance rate of 78%. Hierarchical clustering analysis of transcript signatures showed that the *Iqgap2^−/−^* HCC tumors best co-clustered with advanced T3 human HCC tumors.

A direct mouse-to-human gene expression signature comparison of the animal models of hepatocellular carcinoma is an emerging field with several comprehensive studies published in the past decade. The Thorgeirsson group pioneered the approach in 2004 by conducting an immense mouse-to-human gene signature comparison of 7 different mouse HCC models and 91 human HCC specimens [11]. In this study, the transgenic mouse model simultaneously overexpressing two oncogenes, *Myc* and *Tgfa*, was shown to best recapitulate the molecular signature of aggressive HCC in patients. A more recent study involving a transgenic mouse model overexpressing another known HCC-related oncogene, *c-Met*, demonstrated that hepatic tumors from these mice most relate to patient HCC tumors with activated Wnt/β-catenin signaling pathway [45], a trend observed in our *Iqgap2^−/−^*mice as well. Global transcriptome comparative studies of HCC knockout mouse models include an *Rb* genes triple knockout [46], a DEN-induced HCC model using *Klf6^+/−^* mice [47], and a sirtuin 6 (*Sirt6*) knockout model [48], all three shown to mimic aggressive HCC in humans. The *Sirt6^−/−^* model may be of particular relevance to our study, because, similarly to *Iqgap2^−/−^* mice, its HCC gene signature included vast overexpression of *Afp*, and *Sirt6^−/−^* mice also displayed a distinct metabolic phenotype which included hypoglycemia and increase fat deposition. Lastly, HCC tumors in methionine adenosyltransferase 1A (*MATA1*)-deficient mice were characterized by the impairment of both glucose and fatty acid metabolic pathways [49]. Further comparison of the HCC gene signatures of the *Iqgap2^−/−^* mouse and these models may reveal novel pathway crosstalks and interactions underlying HCC development.

In conclusion, this study comprehensively characterized the gene expression profile of a novel HCC knockout mouse model deficient of a putative tumor suppressor IQGAP2. The gene signatures of *Iqgap2^−/−^* HCC will help unravel the multifaceted mechanisms behind HCC inception and development. The *Iqgap2^−/−^* HCC model was found to closely recapitulate many molecular signatures of human HCC, demonstrating strong predictive power of the identified set of mouse ortholog genes for the human disease. This also validates the use of the *Iqgap2^−/−^* mouse for future testing of HCC therapeutic modalities.

## Supporting Information

Table S1 **Genes differentially expressed between the age of 6 and 24 months in KO livers only.**
(DOCX)

Table S2 **Genes differentially expressed between WT (tumor-free) and KO (HCC) livers from mice of the 24-month-old age group.** This subset of genes represents a “transcript signature” of HCC in the *Iqgap2^−/−^* mouse model.
(DOCX)

Table S3 **Common orthologous genes between the** ***Iqgap2^−/−^*** **HCC and the human GSE6222 microarray data sets.**
(DOCX)
