# Supplementary material for: Transcript Profiling Identifies Iqgap2−/− Mouse as a Model for Advanced Human Hepatocellular Carcinoma
Source: PLoS One. 2013 Aug 12;8(8):e71826. doi: 10.1371/journal.pone.0071826 (PMC3741273; doi:10.1371/journal.pone.0071826)
Supplement: Table S1 — Genes differentially expressed between the age of 6 and 24 months in KO livers only. (DOCX) [file pone.0071826.s001.docx]

**Supplemental Table 1**

|  |  |  | **Fold change** |
| --- | --- | --- | --- |
| **Number** | **Gene name** | **Gene Symbol** | **KO 24mos/6mos** |
| 1 | H19 fetal liver mRNA | H19 | 84.377 |
| 2 | ATP-binding cassette, sub-family C (CFTR/MRP), member 4 | Abcc4 | 45.060 |
| 3 | RIKEN cDNA A730054J21 gene | A730054J21Rik | 38.434 |
| 4 | dual oxidase maturation factor 1 | Duoxa1 | 36.703 |
| 5 | solute carrier family 22 (organic cation transporter), member 3 | Slc22a3 | 35.390 |
| 6 | predicted gene 106 | Gm106 | 33.304 |
| 7 | Rhesus blood group-associated B glycoprotein | Rhbg | 33.286 |
| 8 | RIKEN cDNA 1110006E14 gene | 1110006E14Rik | 29.068 |
| 9 | stearoyl-Coenzyme A desaturase 2 | Scd2 | 23.533 |
| 10 | prominin 1 | Prom1 | 20.923 |
| 11 | small proline-rich protein 1A | Sprr1a | 20.472 |
| 12 | calmodulin-like 4 | Calml4 | 19.656 |
| 13 | brain expressed gene 1 | Bex1 | 18.315 |
| 14 | transmembrane protein 178 | Tmem178 | 17.791 |
| 15 | alpha fetoprotein | Afp | 16.527 |
| 16 | aldo-keto reductase family 1, member B7 | Akr1b7 | 16.113 |
| 17 | WNK lysine deficient protein kinase 4 | Wnk4 | 16.018 |
| 18 | carbonic anhydrase 4 | Car4 | 15.741 |
| 19 | ubiquitin D | Ubd | 15.584 |
| 20 | gamma-aminobutyric acid (GABA) A receptor, subunit beta 3 | Gabrb3 | 15.086 |
| 21 | solute carrier family 39 (zinc transporter), member 2 | Slc39a2 | 13.987 |
| 22 | ceroid-lipofuscinosis, neuronal 6 | Cln6 | 13.576 |
| 23 | vanin 1 | Vnn1 | 13.023 |
| 24 | ATPase, H+ transporting, lysosomal V0 subunit D2 | Atp6v0d2 | 12.566 |
| 25 | DNA segment, Chr 17, human D6S56E 5 | D17H6S56E-5 | 12.546 |
| 26 | G protein-coupled receptor 64 | Gpr64 | 12.368 |
| 27 | oligodendrocyte transcription factor 1 | Olig1 | 12.243 |
| 28 | orosomucoid 2 | Orm2 | 12.176 |
| 29 | kinesin family member 26B | Kif26b | 12.074 |
| 30 | naked cuticle 1 homolog (Drosophila) | Nkd1 | 12.059 |
| 31 | villin 1 | Vil1 | 12.047 |
| 32 | maternally expressed 3 | Meg3 | 12.046 |
| 33 | nuclear protein 1 | Nupr1 | 11.708 |
| 34 | solute carrier family 1 (glutamate/neutral amino acid transporter), member 4 | Slc1a4 | 11.464 |
| 35 | glutathione S-transferase, mu 2 | Gstm2 | 10.964 |
| 36 | RIKEN cDNA 9030619P08 gene | 9030619P08Rik | 10.956 |
| 37 | nemo like kinase | Nlk | 10.942 |
| 38 | carbonic anhydrase 2 | Car2 | 10.783 |
| 39 | RNA imprinted and accumulated in nucleus | Rian | 10.766 |
| 40 | B-cell linker | Blnk | 10.710 |
| 41 | trans-acting transcription factor 5 | Sp5 | 10.669 |
| 42 | dual oxidase 1 | Duox1 | 10.605 |
| 43 | neuronal pentraxin 1 | Nptx1 | 10.471 |
| 44 | tubulointerstitial nephritis antigen | Tinag | 10.373 |
| 45 | solute carrier family 1 (glial high affinity glutamate transporter), member 2 | Slc1a2 | 9.964 |
| 46 | ADAMTS-like 4 | Adamtsl4 | 9.828 |
| 47 | immediate early response 3 | Ier3 | 9.728 |
| 48 | solute carrier family 7 (cationic amino acid transporter, y+ system), member 9 | Slc7a9 | 9.519 |
| 49 | protease, serine, 8 (prostasin) | Prss8 | 9.513 |
| 50 | Wnt inhibitory factor 1 | Wif1 | 9.178 |
| 51 | RIKEN cDNA B830012L14 gene | B830012L14Rik | 9.114 |
| 52 | EGF-like-domain, multiple 6 | Egfl6 | 8.998 |
| 53 | regulator of calcineurin 2 | Rcan2 | 8.800 |
| 54 | delta/notch-like EGF-related receptor | Dner | 8.110 |
| 55 | ELOVL family member 7, elongation of long chain fatty acids (yeast) | Elovl7 | 8.085 |
| 56 | RIKEN cDNA 2200001I15 gene | 2200001I15Rik | 8.058 |
| 57 | pyruvate dehydrogenase kinase, isoenzyme 4 | Pdk4 | 7.770 |
| 58 | protocadherin 24 | Pcdh24 | 7.674 |
| 59 | macrophage activation 2 like | Mpa2l | 7.637 |
| 60 | proteinase 3 | Prtn3 | 7.632 |
| 61 | NAD(P)H dehydrogenase, quinone 1 | Nqo1 | 7.523 |
| 62 | retinol dehydrogenase 9 | Rdh9 | 7.373 |
| 63 | monoacylglycerol O-acyltransferase 2 | Mogat2 | 7.188 |
| 64 | antigen identified by monoclonal antibody Ki 67 | Mki67 | 7.094 |
| 65 | fibroblast growth factor 21 | Fgf21 | 7.087 |
| 66 | S100 calcium binding protein A6 (calcyclin) | S100a6 | 7.061 |
| 67 | monoamine oxidase A | Maoa | 7.032 |
| 68 | protein kinase, cGMP-dependent, type II | Prkg2 | 6.902 |
| 69 | cyclin B2 | Ccnb2 | 6.844 |
| 70 | interferon, alpha-inducible protein 27 like 2B | Ifi27l2b | 6.815 |
| 71 | peroxisome proliferator activated receptor gamma | Pparg | 6.714 |
| 72 | kelch-like 23 (Drosophila) | Klhl23 | 6.679 |
| 73 | lipoprotein lipase | Lpl | 6.593 |
| 74 | myo-inositol 1-phosphate synthase A1 | Isyna1 | 6.554 |
| 75 | coiled-coil domain containing 120 | Ccdc120 | 6.477 |
| 76 | platelet-derived growth factor, C polypeptide | Pdgfc | 6.426 |
| 77 | cDNA sequence BC046404 | BC046404 | 6.322 |
| 78 | S100 calcium binding protein A11 (calgizzarin) | S100a11 | 6.234 |
| 79 | dual-specificity tyrosine-(Y)-phosphorylation regulated kinase 3 | Dyrk3 | 6.218 |
| 80 | glutamate-ammonia ligase (glutamine synthetase) | Glul | 5.994 |
| 81 | suppressor of cytokine signaling 3 | Socs3 | 5.979 |
| 82 | family with sequence similarity 102, member A | Fam102a | 5.958 |
| 83 | ubiquitin-conjugating enzyme E2C | Ube2c | 5.904 |
| 84 | ATPase, H+ transporting, lysosomal V0 subunit E2 | Atp6v0e2 | 5.876 |
| 85 | RIKEN cDNA 2010003K11 gene | 2010003K11Rik | 5.815 |
| 86 | adrenergic receptor, beta 2 | Adrb2 | 5.803 |
| 87 | nuclear factor, erythroid derived 2 | Nfe2 | 5.738 |
| 88 | nucleosome assembly protein 1-like 3 | Nap1l3 | 5.698 |
| 89 | collagen, type VIII, alpha 1 | Col8a1 | 5.539 |
| 90 | patatin-like phospholipase domain containing 3 | Pnpla3 | 5.521 |
| 91 | secreted frizzled-related protein 2 | Sfrp2 | 5.435 |
| 92 | RIKEN cDNA 1700112E06 gene | 1700112E06Rik | 5.400 |
| 93 | coiled-coil domain containing 85B | Ccdc85b | 5.385 |
| 94 | baculoviral IAP repeat-containing 5 | Birc5 | 5.375 |
| 95 | glutathione S-transferase, mu 3 | Gstm3 | 5.332 |
| 96 | camello-like 4 | Cml4 | 5.328 |
| 97 | ATPase, class V, type 10A | Atp10a | 5.302 |
| 98 | GRINL1A complex locus | Gcom1 | 5.300 |
| 99 | carbonyl reductase 3 | Cbr3 | 5.252 |
| 100 | endonuclease domain containing 1 | Endod1 | 5.244 |
| 101 | ect2 oncogene | Ect2 | 5.176 |
| 102 | retinol binding protein 1, cellular | Rbp1 | 5.145 |
| 103 | arginine vasopressin receptor 1A | Avpr1a | 5.139 |
| 104 | RIKEN cDNA B930041F14 gene | B930041F14Rik | 5.078 |
| 105 | antisense Igf2r RNA | Airn | 5.065 |
| 106 | glycerophosphodiester phosphodiesterase domain containing 3 | Gdpd3 | 5.047 |
| 107 | ajuba | Jub | 5.042 |
| 108 | folate hydrolase | Folh1 | 5.037 |
| 109 | RIKEN cDNA 6330417G04 gene | 6330417G04Rik | 5.019 |
| 110 | tubulin, alpha 8 | Tuba8 | 5.015 |
| 111 | GIPC PDZ domain containing family, member 2 | Gipc2 | 5.013 |
| 112 | phosphoserine aminotransferase 1 | Psat1 | 4.991 |
| 113 | RIKEN cDNA D630004K10 gene | D630004K10Rik | 4.944 |
| 114 | tumor necrosis factor receptor superfamily, member 19 | Tnfrsf19 | 4.916 |
| 115 | nuclear paraspeckle assembly transcript 1 (non-protein coding) | Neat1 | 4.868 |
| 116 | axin2 | Axin2 | 4.808 |
| 117 | neural proliferation, differentiation and control gene 1 | Npdc1 | 4.793 |
| 118 | Purkinje cell protein 4-like 1 | Pcp4l1 | 4.772 |
| 119 | coiled-coil domain containing 25 | Ccdc25 | 4.712 |
| 120 | stathmin 1 | Stmn1 | 4.677 |
| 121 | cyclin D1 | Ccnd1 | 4.670 |
| 122 | serine (or cysteine) peptidase inhibitor, clade A (antitrypsin), member 7 | Serpina7 | 4.511 |
| 123 | annexin A5 | Anxa5 | 4.435 |
| 124 | platelet derived growth factor, alpha | Pdgfa | 4.423 |
| 125 | acyl-CoA thioesterase 9 | Acot9 | 4.382 |
| 126 | leucine rich repeat containing G protein coupled receptor 5 | Lgr5 | 4.373 |
| 127 | cytoplasmic polyadenylation element binding protein 1 | Cpeb1 | 4.369 |
| 128 | prokineticin receptor 2 | Prokr2 | 4.355 |
| 129 | pre B-cell leukemia transcription factor 3 | Pbx3 | 4.278 |
| 130 | histone cluster 1, H1c | Hist1h1c | 4.259 |
| 131 | Holliday junction recognition protein | Hjurp | 4.238 |
| 132 | transmembrane protein 136 | Tmem136 | 4.225 |
| 133 | very low density lipoprotein receptor | Vldlr | 4.205 |
| 134 | chemokine (C-X-C motif) ligand 10 | Cxcl10 | 4.179 |
| 135 | biliverdin reductase B (flavin reductase (NADPH)) | Blvrb | 4.152 |
| 136 | guanylate binding protein 2 | Gbp2 | 4.130 |
| 137 | family with sequence similarity 55, member B | Fam55b | 4.117 |
| 138 | expressed sequence AU015536 | AU015536 | 4.072 |
| 139 | cDNA sequence BC037703 | BC037703 | 4.043 |
| 140 | intercellular adhesion molecule 1 | Icam1 | 4.008 |
| 141 | scavenger receptor class A, member 5 (putative) | Scara5 | 3.994 |
| 142 | RIKEN cDNA 2310007H09 gene | 2310007H09Rik | 3.963 |
| 143 | ADP-ribosylation factor-like 2 binding protein | Arl2bp | 3.959 |
| 144 | tetratricopeptide repeat domain 39A | Ttc39a | 3.958 |
| 145 | plasminogen activator, tissue | Plat | 3.935 |
| 146 | RIKEN cDNA 2310021H06 gene | 2310021H06Rik | 3.925 |
| 147 | solute carrier family 13 (sodium-dependent dicarboxylate transporter), member 3 | Slc13a3 | 3.921 |
| 148 | secreted and transmembrane 1A | Sectm1a | 3.893 |
| 149 | 3-hydroxy-3-methylglutaryl-Coenzyme A synthase 1 | Hmgcs1 | 3.893 |
| 150 | transmembrane 4 L six family member 20 | Tm4sf20 | 3.850 |
| 151 | RIKEN cDNA 1700007F19 gene | 1700007F19Rik | 3.826 |
| 152 | CD44 antigen | Cd44 | 3.823 |
| 153 | LIM homeobox protein 6 | Lhx6 | 3.801 |
| 154 | expressed sequence C85403 | C85403 | 3.798 |
| 155 | camello-like 5 | Cml5 | 3.752 |
| 156 | mevalonate (diphospho) decarboxylase | Mvd | 3.744 |
| 157 | glucosidase beta 2 | Gba2 | 3.728 |
| 158 | histone cluster 2, H3c2 | Hist2h3c2 | 3.669 |
| 159 | heat shock protein 2 | Hspa2 | 3.634 |
| 160 | activating transcription factor 3 | Atf3 | 3.624 |
| 161 | fatty acid binding protein 5, epidermal | Fabp5 | 3.579 |
| 162 | bone morphogenetic protein 7 | Bmp7 | 3.570 |
| 163 | methionine adenosyltransferase II, alpha | Mat2a | 3.537 |
| 164 | CD68 antigen | Cd68 | 3.530 |
| 165 | cysteine and glycine-rich protein 2 | Csrp2 | 3.469 |
| 166 | RIKEN cDNA 5830443L24 gene | 5830443L24Rik | 3.466 |
| 167 | histone cluster 2, H2be | Hist2h2be | 3.466 |
| 168 | glycyl-tRNA synthetase | Gars | 3.435 |
| 169 | sulfiredoxin 1 homolog (S. cerevisiae) | Srxn1 | 3.421 |
| 170 | cell division cycle 20 homolog (S. cerevisiae) | Cdc20 | 3.406 |
| 171 | acyl-CoA synthetase long-chain family member 3 | Acsl3 | 3.353 |
| 172 | CD2-associated protein | Cd2ap | 3.352 |
| 173 | heat shock transcription factor 2 binding protein | Hsf2bp | 3.341 |
| 174 | lymphocyte antigen 6 complex, locus D | Ly6d | 3.329 |
| 175 | ankyrin repeat domain 56 | Ankrd56 | 3.315 |
| 176 | 6-phosphofructo-2-kinase/fructose-2,6-biphosphatase 1 | Pfkfb1 | 3.315 |
| 177 | RIKEN cDNA D830012I24 gene | D830012I24Rik | 3.315 |
| 178 | myelin protein zero-like 1 | Mpzl1 | 3.285 |
| 179 | T-box 3 | Tbx3 | 3.267 |
| 180 | cystatin B | Cstb | 3.266 |
| 181 | vesicular, overexpressed in cancer, prosurvival protein 1 | Vopp1 | 3.251 |
| 182 | Indian hedgehog | Ihh | 3.222 |
| 183 | PDZ binding kinase | Pbk | 3.219 |
| 184 | gulonolactone (L-) oxidase | Gulo | 3.209 |
| 185 | growth arrest-specific 2 like 3 | Gas2l3 | 3.208 |
| 186 | neuroepithelial cell transforming gene 1 | Net1 | 3.193 |
| 187 | transcription factor 7, T-cell specific | Tcf7 | 3.188 |
| 188 | interferon gamma inducible protein 47 | Ifi47 | 3.155 |
| 189 | major facilitator superfamily domain containing 7C | Mfsd7c | 3.145 |
| 190 | roundabout homolog 1 (Drosophila) | Robo1 | 3.144 |
| 191 | receptor accessory protein 1 | Reep1 | 3.142 |
| 192 | acetyl-Coenzyme A acetyltransferase 2 | Acat2 | 3.097 |
| 193 | transmembrane protease, serine 2 | Tmprss2 | 3.073 |
| 194 | toll-like receptor 1 | Tlr1 | 3.068 |
| 195 | acyl-CoA synthetase long-chain family member 4 | Acsl4 | 3.053 |
| 196 | collectin sub-family member 12 | Colec12 | 3.017 |
| 197 | cyclin A2 | Ccna2 | 3.016 |
| 198 | protein tyrosine phosphatase, non-receptor type 9 | Ptpn9 | 3.012 |
| 199 | deoxynucleotidyltransferase, terminal | Dntt | 3.007 |
| 200 | phosphomannomutase 1 | Pmm1 | 3.002 |
| 201 | receptor (calcitonin) activity modifying protein 2 | Ramp2 | 0.333 |
| 202 | brain expressed myelocytomatosis oncogene | Bmyc | 0.332 |
| 203 | laminin, alpha 1 | Lama1 | 0.332 |
| 204 | transmembrane protein 184C | Tmem184c | 0.331 |
| 205 | nuclear receptor subfamily 1, group D, member 1 | Nr1d1 | 0.330 |
| 206 | aquaporin 8 | Aqp8 | 0.330 |
| 207 | ornithine transcarbamylase | Otc | 0.329 |
| 208 | LIM homeobox protein 2 | Lhx2 | 0.329 |
| 209 | glutathione S-transferase, theta 3 | Gstt3 | 0.328 |
| 210 | RIKEN cDNA 0610008F07 gene | 0610008F07Rik | 0.327 |
| 211 | tyrosine kinase with immunoglobulin-like and EGF-like domains 1 | Tie1 | 0.327 |
| 212 | RIKEN cDNA 1700012D01 gene | 1700012D01Rik | 0.325 |
| 213 | cytochrome P450, family 4, subfamily a, polypeptide 14 | Cyp4a14 | 0.324 |
| 214 | insulin-like growth factor binding protein 5 | Igfbp5 | 0.324 |
| 215 | synapse defective 1, Rho GTPase, homolog 2 (C. elegans) | Syde2 | 0.322 |
| 216 | peroxisomal membrane protein 2 | Pxmp2 | 0.322 |
| 217 | zinc finger protein 750 | Zfp750 | 0.321 |
| 218 | CD1d1 antigen | Cd1d1 | 0.320 |
| 219 | reversion-inducing-cysteine-rich protein with kazal motifs | Reck | 0.320 |
| 220 | indolethylamine N-methyltransferase | Inmt | 0.318 |
| 221 | RIKEN cDNA 5730414N17 gene | 5730414N17Rik | 0.317 |
| 222 | eukaryotic translation initiation factor 2, subunit 3, structural gene Y-linked | Eif2s3y | 0.315 |
| 223 | DNA segment, Chr 4, Brigham & Women's Genetics 0951 expressed | D4Bwg0951e | 0.315 |
| 224 | cytoplasmic polyadenylation element binding protein 2 | Cpeb2 | 0.312 |
| 225 | ABI gene family, member 3 (NESH) binding protein | Abi3bp | 0.312 |
| 226 | RNA binding motif, single stranded interacting protein | Rbms3 | 0.312 |
| 227 | mannose receptor, C type 1 | Mrc1 | 0.311 |
| 228 | ATP-binding cassette, sub-family C (CFTR/MRP), member 9 | Abcc9 | 0.306 |
| 229 | nicotinamide N-methyltransferase | Nnmt | 0.305 |
| 230 | bone morphogenetic protein 2 | Bmp2 | 0.303 |
| 231 | potassium channel, subfamily K, member 5 | Kcnk5 | 0.302 |
| 232 | phospholipase C-like 2 | Plcl2 | 0.302 |
| 233 | plexin C1 | Plxnc1 | 0.301 |
| 234 | chemokine (C-C motif) ligand 9 | Ccl9 | 0.295 |
| 235 | growth arrest specific 1 | Gas1 | 0.293 |
| 236 | START domain containing 8 | Stard8 | 0.292 |
| 237 | protein-tyrosine sulfotransferase 1 | Tpst1 | 0.290 |
| 238 | transmembrane protein 204 | Tmem204 | 0.286 |
| 239 | ST3 beta-galactoside alpha-2,3-sialyltransferase 5 | St3gal5 | 0.285 |
| 240 | RasGEF domain family, member 1B | Rasgef1b | 0.283 |
| 241 | uroplakin 3B | Upk3b | 0.282 |
| 242 | kinase insert domain protein receptor | Kdr | 0.281 |
| 243 | RIKEN cDNA 4933438K21 gene | 4933438K21Rik | 0.279 |
| 244 | G protein-coupled receptor 182 | Gpr182 | 0.279 |
| 245 | growth differentiation factor 2 | Gdf2 | 0.277 |
| 246 | kynureninase (L-kynurenine hydrolase) | Kynu | 0.275 |
| 247 | extracellular matrix protein 1 | Ecm1 | 0.275 |
| 248 | angiopoietin-like 6 | Angptl6 | 0.275 |
| 249 | cell adhesion molecule 4 | Cadm4 | 0.272 |
| 250 | solute carrier family 13 (sodium-dependent dicarboxylate transporter), member 2 | Slc13a2 | 0.272 |
| 251 | leucine rich repeat containing 16A | Lrrc16a | 0.271 |
| 252 | solute carrier organic anion transporter family, member 1a4 | Slco1a4 | 0.269 |
| 253 | mitochondrial carrier triple repeat 1 | Mcart1 | 0.267 |
| 254 | expressed sequence AI317395 | AI317395 | 0.265 |
| 255 | SET and MYND domain containing 2 | Smyd2 | 0.264 |
| 256 | inhibitor of DNA binding 3 | Id3 | 0.262 |
| 257 | regulator of G-protein signaling 5 | Rgs5 | 0.261 |
| 258 | complement component 8, beta polypeptide | C8b | 0.260 |
| 259 | proline arginine-rich end leucine-rich repeat | Prelp | 0.260 |
| 260 | syntrophin, gamma 2 | Sntg2 | 0.259 |
| 261 | hydroxysteroid (17-beta) dehydrogenase 13 | Hsd17b13 | 0.259 |
| 262 | urocanase domain containing 1 | Uroc1 | 0.257 |
| 263 | tubulointerstitial nephritis antigen-like 1 | Tinagl1 | 0.256 |
| 264 | catenin beta interacting protein 1 | Ctnnbip1 | 0.254 |
| 265 | RIKEN cDNA 2410024N18 gene | 2410024N18Rik | 0.252 |
| 266 | dual specificity phosphatase 1 | Dusp1 | 0.251 |
| 267 | biglycan | Bgn | 0.250 |
| 268 | cytochrome P450, family 2, subfamily d, polypeptide 13 | Cyp2d13 | 0.249 |
| 269 | indoleamine 2,3-dioxygenase 2 | Ido2 | 0.249 |
| 270 | myosin regulatory light chain interacting protein | Mylip | 0.248 |
| 271 | carboxylesterase 6 | Ces6 | 0.248 |
| 272 | cystathionase (cystathionine gamma-lyase) | Cth | 0.246 |
| 273 | energy homeostasis associated | Enho | 0.244 |
| 274 | similar to dHand protein | LOC100046086 | 0.242 |
| 275 | DNA segment, Chr 4, ERATO Doi 298, expressed | D4Ertd298e | 0.240 |
| 276 | serum deprivation response | Sdpr | 0.239 |
| 277 | hydroxysteroid 11-beta dehydrogenase 1 | Hsd11b1 | 0.237 |
| 278 | fibroblast growth factor receptor 2 | Fgfr2 | 0.236 |
| 279 | interferon induced transmembrane protein 1 | Ifitm1 | 0.236 |
| 280 | potassium intermediate/small conductance calcium-activated channel, subfamily N, m 2 | Kcnn2 | 0.234 |
| 281 | peptidylglycine alpha-amidating monooxygenase | Pam | 0.228 |
| 282 | oncoprotein induced transcript 3 | Oit3 | 0.227 |
| 283 | membrane-spanning 4-domains, subfamily A, member 4D | Ms4a4d | 0.224 |
| 284 | RIKEN cDNA 1600002H07 gene | 1600002H07Rik | 0.222 |
| 285 | EH-domain containing 3 | Ehd3 | 0.221 |
| 286 | family with sequence similarity 46, member A | Fam46a | 0.219 |
| 287 | cytochrome P450, family 4, subfamily b, polypeptide 1 | Cyp4b1 | 0.218 |
| 288 | lumican | Lum | 0.218 |
| 289 | superoxide dismutase 3, extracellular | Sod3 | 0.217 |
| 290 | endothelial-specific receptor tyrosine kinase | Tek | 0.212 |
| 291 | retinol saturase (all trans retinol 13,14 reductase) | Retsat | 0.212 |
| 292 | deiodinase, iodothyronine, type I | Dio1 | 0.211 |
| 293 | complement component 9 | C9 | 0.209 |
| 294 | aquaporin 1 | Aqp1 | 0.207 |
| 295 | RIKEN cDNA 3110049J23 gene | 3110049J23Rik | 0.207 |
| 296 | apolipoprotein A-V | Apoa5 | 0.207 |
| 297 | septin 4 | Sept4 | 0.204 |
| 298 | RIKEN cDNA 9230104K21 gene | 9230104K21Rik | 0.203 |
| 299 | inhibitor of DNA binding 1 | Id1 | 0.203 |
| 300 | kelch-like 13 (Drosophila) | Klhl13 | 0.201 |
| 301 | lipoma HMGIC fusion partner | Lhfp | 0.199 |
| 302 | collectin sub-family member 11 | Colec11 | 0.197 |
| 303 | RIKEN cDNA 9130208E07 gene | 9130208E07Rik | 0.195 |
| 304 | alanine-glyoxylate aminotransferase | Agxt | 0.194 |
| 305 | SCO cytochrome oxidase deficient homolog 2 (yeast) | Sco2 | 0.193 |
| 306 | meningioma 1 | Mn1 | 0.191 |
| 307 | RIKEN cDNA 1810046K07 gene | 1810046K07Rik | 0.191 |
| 308 | serine (or cysteine) peptidase inhibitor, clade A, member 3K | Serpina3k | 0.190 |
| 309 | solute carrier family 22 (organic anion transporter), member 7 | Slc22a7 | 0.188 |
| 310 | crystallin, lambda 1 | Cryl1 | 0.185 |
| 311 | major urinary protein 3 | Mup3 | 0.185 |
| 312 | connective tissue growth factor | Ctgf | 0.183 |
| 313 | guanylate cyclase 1, soluble, beta 3 | Gucy1b3 | 0.183 |
| 314 | D site albumin promoter binding protein | Dbp | 0.181 |
| 315 | CD163 antigen | Cd163 | 0.180 |
| 316 | phosphoenolpyruvate carboxykinase 1, cytosolic | Pck1 | 0.178 |
| 317 | expressed sequence AI132487 | AI132487 | 0.176 |
| 318 | Ca2+-dependent activator protein for secretion 2 | Cadps2 | 0.173 |
| 319 | solute carrier family 7 (cationic amino acid transporter, y+ system), member 2 | Slc7a2 | 0.173 |
| 320 | dpy-19-like 3 (C. elegans) | Dpy19l3 | 0.173 |
| 321 | fragile histidine triad gene | Fhit | 0.172 |
| 322 | TRH-degrading enzyme | Trhde | 0.166 |
| 323 | growth arrest specific 2 | Gas2 | 0.164 |
| 324 | apolipoprotein M | Apom | 0.163 |
| 325 | carbonic anhydrase 14 | Car14 | 0.163 |
| 326 | MAM domain containing 2 | Mamdc2 | 0.154 |
| 327 | thioredoxin interacting protein | Txnip | 0.154 |
| 328 | v-maf musculoaponeurotic fibrosarcoma oncogene family, protein B (avian) | Mafb | 0.153 |
| 329 | DEAD (Asp-Glu-Ala-Asp) box polypeptide 3, Y-linked | Ddx3y | 0.148 |
| 330 | formiminotransferase cyclodeaminase | Ftcd | 0.143 |
| 331 | myomesin 2 | Myom2 | 0.143 |
| 332 | esterase 22 | Es22 | 0.140 |
| 333 | epidermal growth factor-containing fibulin-like extracellular matrix protein 1 | Efemp1 | 0.137 |
| 334 | RIKEN cDNA 2810007J24 gene | 2810007J24Rik | 0.134 |
| 335 | carbamoyl-phosphate synthetase 1 | Cps1 | 0.132 |
| 336 | doublecortin-like kinase 3 | Dclk3 | 0.131 |
| 337 | cytochrome c oxidase subunit VIb polypeptide 2 | Cox6b2 | 0.128 |
| 338 | solute carrier family 30, member 10 | Slc30a10 | 0.127 |
| 339 | Kruppel-like factor 10 | Klf10 | 0.124 |
| 340 | hydroxysteroid (17-beta) dehydrogenase 6 | Hsd17b6 | 0.119 |
| 341 | ADAM-like, decysin 1 | Adamdec1 | 0.112 |
| 342 | serine dehydratase-like | Sdsl | 0.106 |
| 343 | cytochrome P450, family 2, subfamily b, polypeptide 10 | Cyp2b10 | 0.106 |
| 344 | solute carrier organic anion transporter family, member 1a1 | Slco1a1 | 0.104 |
| 345 | insulin-like growth factor binding protein 3 | Igfbp3 | 0.102 |
| 346 | collectin sub-family member 10 | Colec10 | 0.094 |
| 347 | cadherin 1 | Cdh1 | 0.088 |
| 348 | RIKEN cDNA B230114P17 gene | B230114P17Rik | 0.087 |
| 349 | cytochrome P450, family 8, subfamily b, polypeptide 1 | Cyp8b1 | 0.086 |
| 350 | cDNA sequence BC089597 | BC089597 | 0.081 |
| 351 | cysteine conjugate-beta lyase 2 | Ccbl2 | 0.079 |
| 352 | ribosomal protein S4, Y-linked 2 | Rps4y2 | 0.054 |
| 353 | predicted gene 10567 | Gm10567 | 0.052 |
| 354 | hepcidin antimicrobial peptide | Hamp | 0.047 |
| 355 | insulin-like growth factor binding protein 2 | Igfbp2 | 0.047 |
| 356 | hepcidin antimicrobial peptide 2 | Hamp2 | 0.047 |
| 357 | glutamate oxaloacetate transaminase 1, soluble | Got1 | 0.037 |
| 358 | 3-hydroxybutyrate dehydrogenase, type 2 | Bdh2 | 0.035 |
| 359 | predicted gene 4848 | Gm4848 | 0.034 |
| 360 | amidohydrolase domain containing 1 | Amdhd1 | 0.028 |
| 361 | arginase, liver | Arg1 | 0.026 |
| 362 | glycine decarboxylase | Gldc | 0.026 |
| 363 | sideroflexin 1 | Sfxn1 | 0.025 |
| 364 | major facilitator superfamily domain containing 2 | Mfsd2 | 0.024 |
| 365 | aldehyde dehydrogenase 1 family, member B1 | Aldh1b1 | 0.021 |
| 366 | alanine-glyoxylate aminotransferase 2-like 1 | Agxt2l1 | 0.021 |
| 367 | asparaginase homolog (S. cerevisiae) | Aspg | 0.020 |
| 368 | serine dehydratase | Sds | 0.016 |
| 369 | histidine ammonia lyase | Hal | 0.008 |
| 370 | glutaminase 2 (liver, mitochondrial) | Gls2 | 0.007 |
| 371 | cytochrome P450, family 2, subfamily f, polypeptide 2 | Cyp2f2 | 0.004 |
