# Supplementary material for: Transcript Profiling Identifies Iqgap2−/− Mouse as a Model for Advanced Human Hepatocellular Carcinoma
Source: PLoS One. 2013 Aug 12;8(8):e71826. doi: 10.1371/journal.pone.0071826 (PMC3741273; doi:10.1371/journal.pone.0071826)
Supplement: Table S2 — Genes differentially expressed between WT (tumor-free) and KO (HCC) livers from mice of the 24-month-old age group. This subset of genes represents a “transcript signature” of HCC in the Iqgap2−/− mouse model. (DOCX) [file pone.0071826.s002.docx]

**Supplemental Table 2**

|  |  |  | **Fold change** |
| --- | --- | --- | --- |
| **Number** | **Gene name** | **Gene Symbol** | **KO24mos/WT24mos** |
| 1 | dual oxidase maturation factor 1 | Duoxa1 | 35.365 |
| 2 | predicted gene 106 | Gm106 | 33.533 |
| 3 | solute carrier family 22 (organic cation transporter), member 3 | Slc22a3 | 33.391 |
| 4 | RIKEN cDNA 1110006E14 gene | 1110006E14Rik | 30.114 |
| 5 | small proline-rich protein 1A | Sprr1a | 19.784 |
| 6 | stearoyl-Coenzyme A desaturase 2 | Scd2 | 17.059 |
| 7 | transmembrane protein 178 | Tmem178 | 15.834 |
| 8 | H19 fetal liver mRNA | H19 | 15.762 |
| 9 | gamma-aminobutyric acid (GABA) A receptor, subunit beta 3 | Gabrb3 | 15.636 |
| 10 | WNK lysine deficient protein kinase 4 | Wnk4 | 15.437 |
| 11 | carbonic anhydrase 4 | Car4 | 15.379 |
| 12 | oligodendrocyte transcription factor 1 | Olig1 | 14.418 |
| 13 | alpha fetoprotein | Afp | 14.183 |
| 14 | miRNA containing gene | Mirg | 13.780 |
| 15 | tubulointerstitial nephritis antigen | Tinag | 13.310 |
| 16 | Rhesus blood group-associated B glycoprotein | Rhbg | 12.875 |
| 17 | ATP-binding cassette, sub-family C (CFTR/MRP), member 4 | Abcc4 | 12.860 |
| 18 | protease, serine, 8 (prostasin) | Prss8 | 12.716 |
| 19 | phosphoglucomutase 5 | Pgm5 | 12.326 |
| 20 | RNA imprinted and accumulated in nucleus | Rian | 12.240 |
| 21 | carbonic anhydrase 2 | Car2 | 12.104 |
| 22 | neuronal pentraxin 1 | Nptx1 | 12.038 |
| 23 | maternally expressed 3 | Meg3 | 11.800 |
| 24 | RIKEN cDNA A730054J21 gene | A730054J21Rik | 11.756 |
| 25 | kinesin family member 26B | Kif26b | 11.287 |
| 26 | G protein-coupled receptor 64 | Gpr64 | 11.244 |
| 27 | dual oxidase 1 | Duox1 | 11.162 |
| 28 | solute carrier family 1 (glutamate/neutral amino acid transporter), member 4 | Slc1a4 | 11.139 |
| 29 | vanin 1 | Vnn1 | 10.997 |
| 30 | trans-acting transcription factor 5 | Sp5 | 10.950 |
| 31 | solute carrier family 7 (cationic amino acid transporter, y+ system), member 9 | Slc7a9 | 10.202 |
| 32 | aldo-keto reductase family 1, member B7 | Akr1b7 | 9.914 |
| 33 | ADAMTS-like 4 | Adamtsl4 | 9.699 |
| 34 | RIKEN cDNA B830012L14 gene | B830012L14Rik | 9.682 |
| 35 | ceroid-lipofuscinosis, neuronal 6 | Cln6 | 9.399 |
| 36 | EGF-like-domain, multiple 6 | Egfl6 | 9.386 |
| 37 | nemo like kinase | Nlk | 9.121 |
| 38 | kelch-like 23 (Drosophila) | Klhl23 | 8.997 |
| 39 | fibroblast growth factor 21 | Fgf21 | 8.892 |
| 40 | retinol dehydrogenase 9 | Rdh9 | 8.871 |
| 41 | delta/notch-like EGF-related receptor | Dner | 8.354 |
| 42 | peroxisome proliferator activated receptor gamma | Pparg | 8.334 |
| 43 | nuclear protein 1 | Nupr1 | 8.250 |
| 44 | naked cuticle 1 homolog (Drosophila) | Nkd1 | 8.176 |
| 45 | ELOVL family member 7, elongation of long chain fatty acids (yeast) | Elovl7 | 8.155 |
| 46 | protein kinase, cGMP-dependent, type II | Prkg2 | 8.137 |
| 47 | ubiquitin D | Ubd | 7.622 |
| 48 | coiled-coil domain containing 25 | Ccdc25 | 7.595 |
| 49 | villin 1 | Vil1 | 7.519 |
| 50 | DNA segment, Chr 17, human D6S56E 5 | D17H6S56E-5 | 7.452 |
| 51 | prominin 1 | Prom1 | 7.393 |
| 52 | solute carrier family 1 (glial high affinity glutamate transporter), member 2 | Slc1a2 | 7.301 |
| 53 | N-acetyltransferase 8 (GCN5-related, putative) | Nat8 | 7.205 |
| 54 | calmodulin-like 4 | Calml4 | 7.177 |
| 55 | ATPase, H+ transporting, lysosomal V0 subunit E2 | Atp6v0e2 | 7.163 |
| 56 | lipoprotein lipase | Lpl | 7.124 |
| 57 | glutathione S-transferase, mu 6 | Gstm6 | 7.122 |
| 58 | monoamine oxidase A | Maoa | 7.117 |
| 59 | very low density lipoprotein receptor | Vldlr | 7.115 |
| 60 | glutathione S-transferase, mu 2 | Gstm2 | 6.836 |
| 61 | monoacylglycerol O-acyltransferase 2 | Mogat2 | 6.772 |
| 62 | cadherin-related family member 2 | Cdhr2 | 6.758 |
| 63 | secreted frizzled-related protein 2 | Sfrp2 | 6.596 |
| 64 | ATPase, H+ transporting, lysosomal V0 subunit D2 | Atp6v0d2 | 6.561 |
| 65 | solute carrier family 39 (zinc transporter), member 2 | Slc39a2 | 6.525 |
| 66 | regulator of calcineurin 2 | Rcan2 | 6.360 |
| 67 | glutathione S-transferase, mu 3 | Gstm3 | 6.151 |
| 68 | cytochrome P450, family 4, subfamily a, polypeptide 12a | Cyp4a12a | 6.105 |
| 69 | Indian hedgehog | Ihh | 6.067 |
| 70 | nuclear factor, erythroid derived 2 | Nfe2 | 6.044 |
| 71 | RIKEN cDNA 2010107G23 gene | 2010107G23Rik | 5.855 |
| 72 | cysteine and glycine-rich protein 2 | Csrp2 | 5.833 |
| 73 | camello-like 5 | Cml5 | 5.749 |
| 74 | collagen, type VIII, alpha 1 | Col8a1 | 5.678 |
| 75 | brain expressed gene 1 | Bex1 | 5.620 |
| 76 | mevalonate (diphospho) decarboxylase | Mvd | 5.594 |
| 77 | antigen identified by monoclonal antibody Ki 67 | Mki67 | 5.479 |
| 78 | cytochrome P450, family 7, subfamily a, polypeptide 1 | Cyp7a1 | 5.422 |
| 79 | family with sequence similarity 102, member A | Fam102a | 5.416 |
| 80 | nucleosome assembly protein 1-like 3 | Nap1l3 | 5.384 |
| 81 | NAD(P)H dehydrogenase, quinone 1 | Nqo1 | 5.344 |
| 82 | GRINL1A complex locus | Gcom1 | 5.332 |
| 83 | tumor necrosis factor receptor superfamily, member 19 | Tnfrsf19 | 5.328 |
| 84 | ATPase, class V, type 10A | Atp10a | 5.256 |
| 85 | RIKEN cDNA 1700112E06 gene | 1700112E06Rik | 5.174 |
| 86 | tubulin, alpha 8 | Tuba8 | 5.147 |
| 87 | carboxypeptidase E | Cpe | 5.135 |
| 88 | baculoviral IAP repeat-containing 5 | Birc5 | 5.084 |
| 89 | coiled-coil domain containing 120 | Ccdc120 | 5.069 |
| 90 | N-myc downstream regulated gene 1 | Ndrg1 | 5.052 |
| 91 | cyclin B2 | Ccnb2 | 5.046 |
| 92 | cytochrome P450, family 2, subfamily c, polypeptide 55 | Cyp2c55 | 5.044 |
| 93 | arginine vasopressin receptor 1A | Avpr1a | 5.032 |
| 94 | ubiquitin-conjugating enzyme E2C | Ube2c | 5.016 |
| 95 | pyruvate dehydrogenase kinase, isoenzyme 4 | Pdk4 | 5.012 |
| 96 | dual-specificity tyrosine-(Y)-phosphorylation regulated kinase 3 | Dyrk3 | 5.008 |
| 97 | GIPC PDZ domain containing family, member 2 | Gipc2 | 4.890 |
| 98 | phospholysine phosphohistidine inorganic pyrophosphate phosphatase | Lhpp | 4.876 |
| 99 | axin2 | Axin2 | 4.873 |
| 100 | coiled-coil domain containing 85B | Ccdc85b | 4.866 |
| 101 | neural proliferation, differentiation and control gene 1 | Npdc1 | 4.849 |
| 102 | RIKEN cDNA D130043K22 gene | D130043K22Rik | 4.843 |
| 103 | Wnt inhibitory factor 1 | Wif1 | 4.642 |
| 104 | tetratricopeptide repeat domain 39A | Ttc39a | 4.630 |
| 105 | RIKEN cDNA B930041F14 gene | B930041F14Rik | 4.571 |
| 106 | patatin-like phospholipase domain containing 3 | Pnpla3 | 4.555 |
| 107 | prokineticin receptor 2 | Prokr2 | 4.485 |
| 108 | LIM domain only 2 | Lmo2 | 4.474 |
| 109 | carbonyl reductase 3 | Cbr3 | 4.450 |
| 110 | acyl-CoA thioesterase 9 | Acot9 | 4.450 |
| 111 | ajuba | Jub | 4.419 |
| 112 | endonuclease domain containing 1 | Endod1 | 4.411 |
| 113 | retinol dehydrogenase 11 | Rdh11 | 4.379 |
| 114 | platelet derived growth factor, alpha | Pdgfa | 4.353 |
| 115 | acyl-CoA thioesterase 1 | Acot1 | 4.346 |
| 116 | macrophage activation 2 like | Mpa2l | 4.344 |
| 117 | arginase type II | Arg2 | 4.340 |
| 118 | B-cell linker | Blnk | 4.319 |
| 119 | major facilitator superfamily domain containing 7C | Mfsd7c | 4.315 |
| 120 | high mobility group box 3 | Hmgb3 | 4.296 |
| 121 | glutathione S-transferase, mu 4 | Gstm4 | 4.294 |
| 122 | leucine rich repeat containing G protein coupled receptor 5 | Lgr5 | 4.226 |
| 123 | histone cluster 2, H3c2, pseudogene | Hist2h3c2-ps | 4.224 |
| 124 | retinol binding protein 1, cellular | Rbp1 | 4.189 |
| 125 | family with sequence similarity 25, member C | Fam25c | 4.182 |
| 126 | platelet-derived growth factor, C polypeptide | Pdgfc | 4.152 |
| 127 | ADP-ribosylation factor-like 2 binding protein | Arl2bp | 4.114 |
| 128 | S100 calcium binding protein A11 (calgizzarin) | S100a11 | 4.113 |
| 129 | plasminogen activator, tissue | Plat | 4.108 |
| 130 | transmembrane 4 L six family member 20 | Tm4sf20 | 4.108 |
| 131 | RIKEN cDNA 1700007F19 gene | 1700007F19Rik | 4.098 |
| 132 | expressed sequence C85403 | C85403 | 4.088 |
| 133 | transmembrane protein 136 | Tmem136 | 4.083 |
| 134 | carboxylesterase 1G | Ces1g | 4.031 |
| 135 | ect2 oncogene | Ect2 | 4.017 |
| 136 | S100 calcium binding protein A6 (calcyclin) | S100a6 | 3.986 |
| 137 | RIKEN cDNA D630039A03 gene | D630039A03Rik | 3.973 |
| 138 | RIKEN cDNA 4930455C21 gene | 4930455C21Rik | 3.926 |
| 139 | claudin 2 | Cldn2 | 3.925 |
| 140 | Purkinje cell protein 4-like 1 | Pcp4l1 | 3.893 |
| 141 | 3-hydroxy-3-methylglutaryl-Coenzyme A synthase 1 | Hmgcs1 | 3.893 |
| 142 | expressed sequence AU015536 | AU015536 | 3.883 |
| 143 | serine (or cysteine) peptidase inhibitor, clade A, member 6 | Serpina6 | 3.834 |
| 144 | glucosidase beta 2 | Gba2 | 3.831 |
| 145 | histone cluster 2, H2be | Hist2h2be | 3.831 |
| 146 | integral membrane protein 2A | Itm2a | 3.760 |
| 147 | mab-21-like 3 (C. elegans) | Mab21l3 | 3.743 |
| 148 | hydroxyacid oxidase 2 | Hao2 | 3.739 |
| 149 | neuron derived neurotrophic factor | Nenf | 3.705 |
| 150 | phosphoserine aminotransferase 1 | Psat1 | 3.682 |
| 151 | receptor accessory protein 1 | Reep1 | 3.567 |
| 152 | cytoplasmic polyadenylation element binding protein 1 | Cpeb1 | 3.547 |
| 153 | histocompatibility 2, Q region locus 1 | H2-Q1 | 3.509 |
| 154 | cathepsin E | Ctse | 3.467 |
| 155 | annexin A5 | Anxa5 | 3.466 |
| 156 | cholinergic receptor, nicotinic, alpha polypeptide 2 (neuronal) | Chrna2 | 3.453 |
| 157 | transmembrane protein 71 | Tmem71 | 3.440 |
| 158 | glutamate-ammonia ligase (glutamine synthetase) | Glul | 3.413 |
| 159 | protein disulfide isomerase-like, testis expressed | Pdilt | 3.405 |
| 160 | exophilin 5 | Exph5 | 3.372 |
| 161 | acetyl-Coenzyme A acetyltransferase 2 | Acat2 | 3.363 |
| 162 | gulonolactone (L-) oxidase | Gulo | 3.359 |
| 163 | reproductive homeobox 5 | Rhox5 | 3.358 |
| 164 | dual specificity phosphatase 14 | Dusp14 | 3.336 |
| 165 | REC8 homolog (yeast) | Rec8 | 3.334 |
| 166 | roundabout homolog 1 (Drosophila) | Robo1 | 3.333 |
| 167 | RIKEN cDNA 2310021H06 gene | 2310021H06Rik | 3.329 |
| 168 | phosphodiesterase 4D, cAMP specific | Pde4d | 3.323 |
| 169 | fatty acid binding protein 5, epidermal | Fabp5 | 3.306 |
| 170 | fat storage-inducing transmembrane protein 1 | Fitm1 | 3.291 |
| 171 | immediate early response 3 | Ier3 | 3.288 |
| 172 | myelin protein zero-like 1 | Mpzl1 | 3.287 |
| 173 | LIM homeobox protein 6 | Lhx6 | 3.276 |
| 174 | secretory carrier membrane protein 5 | Scamp5 | 3.273 |
| 175 | cDNA sequence BC046404 | BC046404 | 3.264 |
| 176 | adrenergic receptor, beta 2 | Adrb2 | 3.250 |
| 177 | neuroepithelial cell transforming gene 1 | Net1 | 3.249 |
| 178 | family with sequence similarity 89, member A | Fam89a | 3.247 |
| 179 | folate hydrolase | Folh1 | 3.239 |
| 180 | cyclin D1 | Ccnd1 | 3.235 |
| 181 | glycogenin | Gyg | 3.229 |
| 182 | cell division cycle 20 homolog (S. cerevisiae) | Cdc20 | 3.226 |
| 183 | RIKEN cDNA 2010003K11 gene | 2010003K11Rik | 3.211 |
| 184 | acyl-CoA synthetase long-chain family member 4 | Acsl4 | 3.206 |
| 185 | tribbles homolog 2 (Drosophila) | Trib2 | 3.204 |
| 186 | glycerophosphodiester phosphodiesterase domain containing 3 | Gdpd3 | 3.203 |
| 187 | CD2-associated protein | Cd2ap | 3.194 |
| 188 | PDZ binding kinase | Pbk | 3.173 |
| 189 | RIKEN cDNA D830012I24 gene | D830012I24Rik | 3.160 |
| 190 | epithelial membrane protein 2 | Emp2 | 3.153 |
| 191 | phosphomannomutase 1 | Pmm1 | 3.149 |
| 192 | cystatin B | Cstb | 3.145 |
| 193 | sulfiredoxin 1 homolog (S. cerevisiae) | Srxn1 | 3.113 |
| 194 | RIKEN cDNA 1110034G24 gene | 1110034G24Rik | 3.104 |
| 195 | aldo-keto reductase family 1, member C20 | Akr1c20 | 3.099 |
| 196 | protein tyrosine phosphatase-like (proline instead of catalytic arginine), member b | Ptplb | 3.092 |
| 197 | myo-inositol 1-phosphate synthase A1 | Isyna1 | 3.083 |
| 198 | hydroxy-delta-5-steroid dehydrogenase, 3 beta- and steroid delta-isomerase 4 | Hsd3b4 | 3.053 |
| 199 | serine incorporator 5 | Serinc5 | 3.031 |
| 200 | WNT1 inducible signaling pathway protein 1 | Wisp1 | 3.024 |
| 201 | ArfGAP with SH3 domain, ankyrin repeat and PH domain 2 | Asap2 | 3.020 |
| 202 | T-box 3 | Tbx3 | 3.016 |
| 203 | peptidylglycine alpha-amidating monooxygenase | Pam | 0.332 |
| 204 | extracellular matrix protein 1 | Ecm1 | 0.332 |
| 205 | cytochrome P450, family 2, subfamily d, polypeptide 13 | Cyp2d13 | 0.331 |
| 206 | RIKEN cDNA C730036E19 gene | C730036E19Rik | 0.331 |
| 207 | carboxylesterase 1E | Ces1e | 0.331 |
| 208 | predicted gene 10567 | Gm10567 | 0.330 |
| 209 | histocompatibility 2, Q region locus 7 | H2-Q7 | 0.330 |
| 210 | argininosuccinate lyase | Asl | 0.329 |
| 211 | RIKEN cDNA 2810007J24 gene | 2810007J24Rik | 0.328 |
| 212 | RIKEN cDNA 6030422H21 gene | 6030422H21Rik | 0.328 |
| 213 | oncoprotein induced transcript 3 | Oit3 | 0.327 |
| 214 | NEDD4 binding protein 2-like 1 | N4bp2l1 | 0.326 |
| 215 | period homolog 1 (Drosophila) | Per1 | 0.325 |
| 216 | SCO cytochrome oxidase deficient homolog 2 (yeast) | Sco2 | 0.322 |
| 217 | decorin | Dcn | 0.322 |
| 218 | tubulointerstitial nephritis antigen-like 1 | Tinagl1 | 0.322 |
| 219 | G protein-coupled receptor 137B, pseudogene | Gpr137b-ps | 0.319 |
| 220 | inhibitor of DNA binding 4 | Id4 | 0.319 |
| 221 | DNA segment, Chr 4, ERATO Doi 298, expressed | D4Ertd298e | 0.317 |
| 222 | musculoskeletal, embryonic nuclear protein 1 | Mustn1 | 0.315 |
| 223 | lipin 1 | Lpin1 | 0.315 |
| 224 | ficolin A | Fcna | 0.314 |
| 225 | chemokine (C-X-C motif) ligand 1 | Cxcl1 | 0.312 |
| 226 | indoleamine 2,3-dioxygenase 2 | Ido2 | 0.312 |
| 227 | CD38 antigen | Cd38 | 0.311 |
| 228 | RIKEN cDNA 5730414N17 gene | 5730414N17Rik | 0.311 |
| 229 | chemokine (C-C motif) ligand 9 | Ccl9 | 0.311 |
| 230 | zinc finger protein 874a | Zfp874a | 0.310 |
| 231 | EH-domain containing 3 | Ehd3 | 0.308 |
| 232 | lipocalin 13 | Lcn13 | 0.306 |
| 233 | prostaglandin E receptor 2 (subtype EP2) | Ptger2 | 0.306 |
| 234 | cryptochrome 1 (photolyase-like) | Cry1 | 0.305 |
| 235 | dihydroxyacetone kinase 2 homolog (yeast) | Dak | 0.304 |
| 236 | cystathionase (cystathionine gamma-lyase) | Cth | 0.304 |
| 237 | gap junction protein, alpha 4 | Gja4 | 0.303 |
| 238 | retinol saturase (all trans retinol 13,14 reductase) | Retsat | 0.302 |
| 239 | eukaryotic translation initiation factor 2, subunit 3, structural gene Y-linked | Eif2s3y | 0.302 |
| 240 | cathepsin C | Ctsc | 0.302 |
| 241 | heparanase | Hpse | 0.302 |
| 242 | transmembrane protein 47 | Tmem47 | 0.301 |
| 243 | SET and MYND domain containing 2 | Smyd2 | 0.301 |
| 244 | RAB30, member RAS oncogene family | Rab30 | 0.300 |
| 245 | growth arrest specific 2 | Gas2 | 0.298 |
| 246 | guanylate cyclase 1, soluble, beta 3 | Gucy1b3 | 0.297 |
| 247 | SPARC related modular calcium binding 2 | Smoc2 | 0.296 |
| 248 | potassium channel, subfamily T, member 2 | Kcnt2 | 0.295 |
| 249 | catenin beta interacting protein 1 | Ctnnbip1 | 0.295 |
| 250 | energy homeostasis associated | Enho | 0.294 |
| 251 | macrophage receptor with collagenous structure | Marco | 0.293 |
| 252 | D site albumin promoter binding protein | Dbp | 0.292 |
| 253 | endothelial-specific receptor tyrosine kinase | Tek | 0.291 |
| 254 | bone morphogenetic protein 2 | Bmp2 | 0.290 |
| 255 | solute carrier family 37 (glucose-6-phosphate transporter), member 4 | Slc37a4 | 0.289 |
| 256 | platelet factor 4 | Pf4 | 0.289 |
| 257 | RIKEN cDNA 1810046K07 gene | 1810046K07Rik | 0.289 |
| 258 | hydroxy-delta-5-steroid dehydrogenase, 3 beta- and steroid delta-isomerase 5 | Hsd3b5 | 0.287 |
| 259 | fatty acid binding protein 7, brain | Fabp7 | 0.286 |
| 260 | RIKEN cDNA 1100001G20 gene | 1100001G20Rik | 0.286 |
| 261 | G protein-coupled receptor 182 | Gpr182 | 0.286 |
| 262 | paternally expressed 3 | Peg3 | 0.286 |
| 263 | expressed sequence AU015263 | AU015263 | 0.286 |
| 264 | RIKEN cDNA 9330161A08 gene | 9330161A08Rik | 0.285 |
| 265 | arrestin, beta 1 | Arrb1 | 0.284 |
| 266 | proline arginine-rich end leucine-rich repeat | Prelp | 0.281 |
| 267 | mannose receptor, C type 1 | Mrc1 | 0.280 |
| 268 | transmembrane protein 184C | Tmem184c | 0.279 |
| 269 | RIKEN cDNA D630033O11 gene | D630033O11Rik | 0.278 |
| 270 | glutamate receptor, metabotropic 8 | Grm8 | 0.276 |
| 271 | solute carrier organic anion transporter family, member 1a1 | Slco1a1 | 0.276 |
| 272 | E2F transcription factor 7 | E2f7 | 0.276 |
| 273 | MAM domain containing 2 | Mamdc2 | 0.275 |
| 274 | phospholipase A2, group VII (platelet-activating factor acetylhydrolase, plasma) | Pla2g7 | 0.272 |
| 275 | WAP four-disulfide core domain 2 | Wfdc2 | 0.271 |
| 276 | ATP-binding cassette, sub-family C (CFTR/MRP), member 9 | Abcc9 | 0.270 |
| 277 | proteoglycan 4 (megakaryocyte stimulating factor, articular superficial zone protein) | Prg4 | 0.268 |
| 278 | hydroxysteroid (17-beta) dehydrogenase 13 | Hsd17b13 | 0.266 |
| 279 | potassium intermediate/small conductance calcium-activated channel, subfamily N, member 2 | Kcnn2 | 0.266 |
| 280 | septin 4 | Sept4 | 0.265 |
| 281 | insulin-like growth factor binding protein 5 | Igfbp5 | 0.265 |
| 282 | regulator of G-protein signaling 5 | Rgs5 | 0.262 |
| 283 | RasGEF domain family, member 1B | Rasgef1b | 0.259 |
| 284 | solute carrier family 7 (cationic amino acid transporter, y+ system), member 2 | Slc7a2 | 0.258 |
| 285 | cytochrome P450, family 2, subfamily j, polypeptide 9 | Cyp2j9 | 0.256 |
| 286 | C-type lectin domain family 1, member b | Clec1b | 0.256 |
| 287 | potassium channel tetramerisation domain containing 12 | Kctd12 | 0.255 |
| 288 | hepcidin antimicrobial peptide 2 | Hamp2 | 0.254 |
| 289 | growth differentiation factor 2 | Gdf2 | 0.253 |
| 290 | myosin regulatory light chain interacting protein | Mylip | 0.252 |
| 291 | lumican | Lum | 0.250 |
| 292 | cytochrome P450, family 4, subfamily b, polypeptide 1 | Cyp4b1 | 0.249 |
| 293 | sulfotransferase family 5A, member 1 | Sult5a1 | 0.248 |
| 294 | cytochrome c oxidase subunit VIb polypeptide 2 | Cox6b2 | 0.248 |
| 295 | crystallin, lambda 1 | Cryl1 | 0.247 |
| 296 | kelch-like 13 (Drosophila) | Klhl13 | 0.246 |
| 297 | formin-like 2 | Fmnl2 | 0.245 |
| 298 | adenylate kinase 4 | Ak4 | 0.241 |
| 299 | hydroxysteroid 11-beta dehydrogenase 1 | Hsd11b1 | 0.241 |
| 300 | dpy-19-like 3 (C. elegans) | Dpy19l3 | 0.239 |
| 301 | hydroxysteroid (17-beta) dehydrogenase 6 | Hsd17b6 | 0.238 |
| 302 | phenazine biosynthesis-like protein domain containing 2 | Pbld2 | 0.237 |
| 303 | biglycan | Bgn | 0.237 |
| 304 | leukocyte cell derived chemotaxin 1 | Lect1 | 0.235 |
| 305 | serum/glucocorticoid regulated kinase 1 | Sgk1 | 0.235 |
| 306 | solute carrier family 30, member 10 | Slc30a10 | 0.234 |
| 307 | plasmalemma vesicle associated protein | Plvap | 0.232 |
| 308 | glutathione S-transferase, theta 3 | Gstt3 | 0.231 |
| 309 | collectin sub-family member 11 | Colec11 | 0.230 |
| 310 | solute carrier family 3, member 1 | Slc3a1 | 0.228 |
| 311 | aquaporin 1 | Aqp1 | 0.225 |
| 312 | serine (or cysteine) peptidase inhibitor, clade A, member 3K | Serpina3k | 0.224 |
| 313 | interferon regulatory factor 7 | Irf7 | 0.223 |
| 314 | v-maf musculoaponeurotic fibrosarcoma oncogene family, protein B (avian) | Mafb | 0.222 |
| 315 | cytochrome P450, family 17, subfamily a, polypeptide 1 | Cyp17a1 | 0.220 |
| 316 | V-set and immunoglobulin domain containing 4 | Vsig4 | 0.219 |
| 317 | superoxide dismutase 3, extracellular | Sod3 | 0.217 |
| 318 | urocanase domain containing 1 | Uroc1 | 0.216 |
| 319 | carbonic anhydrase 14 | Car14 | 0.216 |
| 320 | forkhead box Q1 | Foxq1 | 0.215 |
| 321 | membrane-spanning 4-domains, subfamily A, member 4D | Ms4a4d | 0.214 |
| 322 | lipoma HMGIC fusion partner | Lhfp | 0.213 |
| 323 | mitochondrial carrier triple repeat 1 | Mcart1 | 0.208 |
| 324 | regulator of G-protein signaling 16 | Rgs16 | 0.205 |
| 325 | major urinary protein 10 | Mup10 | 0.203 |
| 326 | arachidonate 5-lipoxygenase activating protein | Alox5ap | 0.202 |
| 327 | solute carrier organic anion transporter family, member 1a4 | Slco1a4 | 0.197 |
| 328 | cytochrome P450, family 8, subfamily b, polypeptide 1 | Cyp8b1 | 0.196 |
| 329 | CD163 antigen | Cd163 | 0.196 |
| 330 | formiminotransferase cyclodeaminase | Ftcd | 0.195 |
| 331 | neuregulin 4 | Nrg4 | 0.195 |
| 332 | C-type lectin domain family 4, member f | Clec4f | 0.194 |
| 333 | interferon induced transmembrane protein 1 | Ifitm1 | 0.191 |
| 334 | apolipoprotein A-V | Apoa5 | 0.189 |
| 335 | serine (or cysteine) peptidase inhibitor, clade A, member 3M | Serpina3m | 0.189 |
| 336 | doublecortin-like kinase 3 | Dclk3 | 0.188 |
| 337 | fragile histidine triad gene | Fhit | 0.183 |
| 338 | lymphocyte antigen 6 complex, locus A | Ly6a | 0.183 |
| 339 | IQ motif containing GTPase activating protein 2 | Iqgap2 | 0.180 |
| 340 | inhibitor of DNA binding 1 | Id1 | 0.177 |
| 341 | Ca2+-dependent activator protein for secretion 2 | Cadps2 | 0.176 |
| 342 | cytochrome P450, family 4, subfamily a, polypeptide 14 | Cyp4a14 | 0.175 |
| 343 | RIKEN cDNA 9230104K21 gene | 9230104K21Rik | 0.172 |
| 344 | phosphoenolpyruvate carboxykinase 1, cytosolic | Pck1 | 0.169 |
| 345 | DEAD (Asp-Glu-Ala-Asp) box polypeptide 3, Y-linked | Ddx3y | 0.168 |
| 346 | insulin-like growth factor binding protein 3 | Igfbp3 | 0.167 |
| 347 | RIKEN cDNA 1600002H07 gene | 1600002H07Rik | 0.165 |
| 348 | inhibitor of DNA binding 3 | Id3 | 0.162 |
| 349 | solute carrier family 25, member 47 | Slc25a47 | 0.158 |
| 350 | dual specificity phosphatase 1 | Dusp1 | 0.158 |
| 351 | metallothionein 1 | Mt1 | 0.157 |
| 352 | thioredoxin interacting protein | Txnip | 0.157 |
| 353 | chemokine (C-X-C motif) ligand 13 | Cxcl13 | 0.152 |
| 354 | WD repeat and SOCS box-containing 1 | Wsb1 | 0.148 |
| 355 | ST3 beta-galactoside alpha-2,3-sialyltransferase 5 | St3gal5 | 0.146 |
| 356 | ADAM-like, decysin 1 | Adamdec1 | 0.146 |
| 357 | serine (or cysteine) peptidase inhibitor, clade A, member 4, pseudogene 1 | Serpina4-ps1 | 0.145 |
| 358 | RIKEN cDNA B230114P17 gene | B230114P17Rik | 0.144 |
| 359 | apolipoprotein M | Apom | 0.142 |
| 360 | solute carrier family 22 (organic anion transporter), member 7 | Slc22a7 | 0.136 |
| 361 | epidermal growth factor-containing fibulin-like extracellular matrix protein 1 | Efemp1 | 0.133 |
| 362 | Kruppel-like factor 10 | Klf10 | 0.131 |
| 363 | carbamoyl-phosphate synthetase 1 | Cps1 | 0.130 |
| 364 | connective tissue growth factor | Ctgf | 0.129 |
| 365 | cell adhesion molecule 4 | Cadm4 | 0.123 |
| 366 | cadherin 1 | Cdh1 | 0.121 |
| 367 | predicted gene 7969 | Gm7969 | 0.121 |
| 368 | metallothionein 2 | Mt2 | 0.119 |
| 369 | family with sequence similarity 46, member A | Fam46a | 0.113 |
| 370 | BMP-binding endothelial regulator | Bmper | 0.112 |
| 371 | collectin sub-family member 10 | Colec10 | 0.100 |
| 372 | ribosomal protein S4, Y-linked 2 | Rps4y2 | 0.098 |
| 373 | deiodinase, iodothyronine, type I | Dio1 | 0.095 |
| 374 | serine dehydratase-like | Sdsl | 0.093 |
| 375 | myomesin 2 | Myom2 | 0.089 |
| 376 | orosomucoid 3 | Orm3 | 0.084 |
| 377 | 3-hydroxybutyrate dehydrogenase, type 2 | Bdh2 | 0.079 |
| 378 | insulin-like growth factor binding protein 2 | Igfbp2 | 0.073 |
| 379 | cytochrome P450, family 2, subfamily b, polypeptide 10 | Cyp2b10 | 0.073 |
| 380 | serine (or cysteine) peptidase inhibitor, clade E, member 2 | Serpine2 | 0.064 |
| 381 | amidohydrolase domain containing 1 | Amdhd1 | 0.063 |
| 382 | nicotinamide N-methyltransferase | Nnmt | 0.062 |
| 383 | alanine-glyoxylate aminotransferase 2-like 1 | Agxt2l1 | 0.062 |
| 384 | hepcidin antimicrobial peptide | Hamp | 0.056 |
| 385 | glutamate oxaloacetate transaminase 1, soluble | Got1 | 0.053 |
| 386 | cysteine conjugate-beta lyase 2 | Ccbl2 | 0.053 |
| 387 | thiosulfate sulfurtransferase (rhodanese)-like domain containing 1 | Tstd1 | 0.045 |
| 388 | cDNA sequence BC089597 | BC089597 | 0.038 |
| 389 | aldehyde dehydrogenase 1 family, member B1 | Aldh1b1 | 0.036 |
| 390 | glycine decarboxylase | Gldc | 0.036 |
| 391 | sideroflexin 1 | Sfxn1 | 0.027 |
| 392 | arginase, liver | Arg1 | 0.025 |
| 393 | sulfotransferase family 3A, member 1 | Sult3a1 | 0.022 |
| 394 | major facilitator superfamily domain containing 2A | Mfsd2a | 0.020 |
| 395 | serine dehydratase | Sds | 0.020 |
| 396 | flavin containing monooxygenase 3 | Fmo3 | 0.020 |
| 397 | glutaminase 2 (liver, mitochondrial) | Gls2 | 0.012 |
| 398 | histidine ammonia lyase | Hal | 0.010 |
| 399 | cytochrome P450, family 2, subfamily f, polypeptide 2 | Cyp2f2 | 0.005 |
