# Supplementary material for: Transcript Profiling Identifies Iqgap2−/− Mouse as a Model for Advanced Human Hepatocellular Carcinoma
Source: PLoS One. 2013 Aug 12;8(8):e71826. doi: 10.1371/journal.pone.0071826 (PMC3741273; doi:10.1371/journal.pone.0071826)
Supplement: Table S3 — Common orthologous genes between the Iqgap2−/− HCC and the human GSE6222 microarray data sets. (DOCX) [file pone.0071826.s003.docx]

**Supplemental table 3**

|  |  | **Mouse** | **Human** |  |  |
| --- | --- | --- | --- | --- | --- |
| **Number** | **Gene Names** | **KO/WT** | **Tumor/Normal** | **Concordance** | |
| 1 | 3-hydroxybutyrate dehydrogenase, type 1 | 1.419 | -1.247 | N |  |
| 2 | acyl-coa synthetase long-chain family member 4 | 1.695 | 1.13 | Y |  |
| 3 | alanine-glyoxylate aminotransferase 2-like 1 | -1.717 | -1.024 | Y |  |
| 4 | aldehyde dehydrogenase 1 family, member b1 | -1.818 | -1.738 | Y |  |
| 5 | aldehyde oxidase 1 | 1.696 | -1.298 | N |  |
| 6 | aldolase b, fructose-bisphosphate | -1.782 | -1.293 | Y |  |
| 7 | amidohydrolase domain containing 1 | -1.773 | -1.325 | Y |  |
| 8 | angiopoietin-like 6 | -1.771 | -2.203 | Y |  |
| 9 | antigen identified by monoclonal antibody ki-67 | 1.61 | 1.491 | Y |  |
| 10 | apolipoprotein a-v | -1.735 | -1.361 | Y |  |
| 11 | arfgap with sh3 domain, ankyrin repeat and ph domain 2 | 1.542 | 1.369 | Y |  |
| 12 | argininosuccinate lyase | -1.658 | -1.605 | Y |  |
| 13 | asparaginase homolog (s. cerevisiae) | -1.811 | -2.147 | Y |  |
| 14 | atp-binding cassette, sub-family a (abc1), member 8 | 1.673 | -1.506 | N |  |
| 15 | baculoviral iap repeat-containing 5 | 1.777 | 1.359 | Y |  |
| 16 | bile acid coenzyme a: amino acid n-acyltransferase (glycine n-choloyltransferase) | -1.768 | -1.234 | Y |  |
| 17 | c-type lectin domain family 1, member b | -1.496 | -2.555 | Y |  |
| 18 | c-type lectin domain family 4, member g | -1.76 | -2.551 | Y |  |
| 19 | ca++-dependent secretion activator 2 | -1.527 | 1.424 | N |  |
| 20 | cadherin 1, type 1, e-cadherin (epithelial) | -1.726 | -1.226 | Y |  |
| 21 | cap, adenylate cyclase-associated protein, 2 (yeast) | 1.294 | 1.633 | Y |  |
| 22 | carbamoyl-phosphate synthetase 1, mitochondrial | -1.654 | -1.09 | Y |  |
| 23 | carbonic anhydrase ii | 1.799 | -1.576 | N |  |
| 24 | cd163 molecule | -1.335 | -1.619 | Y |  |
| 25 | cd1d molecule | -1.741 | -1.553 | Y |  |
| 26 | cell division cycle 2, g1 to s and g2 to m | 1.503 | 1.439 | Y |  |
| 27 | cell division cycle 20 homolog (s. cerevisiae) | 1.758 | 1.576 | Y |  |
| 28 | chemokine (c-x-c motif) ligand 12 (stromal cell-derived factor 1) | -1.792 | -1.813 | Y |  |
| 29 | chemokine (c-x-c motif) ligand 2 | -1.439 | -1.899 | Y |  |
| 30 | chromosome 14 open reading frame 68 | -1.812 | -1.54 | Y |  |
| 31 | chromosome 8 open reading frame 4 | -1.556 | -1.98 | Y |  |
| 32 | chromosome 9 open reading frame 150 | -1.794 | -1.561 | Y |  |
| 33 | collectin sub-family member 10 (c-type lectin) | -1.755 | -2.227 | Y |  |
| 34 | collectin sub-family member 11 | -1.733 | -1.617 | Y |  |
| 35 | complement component 1, q subcomponent, c chain | -0.916 | -1.656 | Y |  |
| 36 | complement component 6 | 1.006 | -1.132 | N |  |
| 37 | complement component 8, beta polypeptide | -1.558 | -1.19 | Y |  |
| 38 | complement component 9 | -0.876 | -1.993 | Y |  |
| 39 | complement factor h-related 2 | -1.671 | -0.822 | Y |  |
| 40 | complement factor properdin | -0.965 | -2.419 | Y |  |
| 41 | crystallin, lambda 1 | -1.753 | -1.018 | Y |  |
| 42 | cut-like homeobox 2 | -1.089 | -1.093 | Y |  |
| 43 | cyclin a2 | 1.661 | 1.118 | Y |  |
| 44 | cyclin b1 | 1.674 | 1.579 | Y |  |
| 45 | cyclin d1 | 1.596 | -1.086 | N |  |
| 46 | cyclin-dependent kinase inhibitor 2c (p18, inhibits cdk4) | 1.78 | 1.6 | Y |  |
| 47 | cystathionase (cystathionine gamma-lyase) | -1.74 | -1.13 | Y |  |
| 48 | cysteine and glycine-rich protein 2 | 1.796 | 1.151 | Y |  |
| 49 | cytochrome p450, family 1, subfamily a, polypeptide 2 | 1.78 | -1.421 | N |  |
| 50 | cytochrome p450, family 2, subfamily b, polypeptide 6 | -1.813 | -2.092 | Y |  |
| 51 | cytochrome p450, family 2, subfamily c, polypeptide 18 | 1.582 | -1.633 | N |  |
| 52 | cytochrome p450, family 2, subfamily c, polypeptide 19 | -1.267 | -2.049 | Y |  |
| 53 | cytochrome p450, family 3, subfamily a, polypeptide 4 | -0.974 | -1.283 | Y |  |
| 54 | cytochrome p450, family 3, subfamily a, polypeptide 7 | -1.762 | -1.09 | Y |  |
| 55 | cytochrome p450, family 4, subfamily a, polypeptide 22 | 1.301 | -1.605 | N |  |
| 56 | cytochrome p450, family 7, subfamily a, polypeptide 1 | 1.435 | 0.532 | Y |  |
| 57 | cytochrome p450, family 8, subfamily b, polypeptide 1 | -1.276 | -0.796 | Y |  |
| 58 | decorin | -1.739 | -2.006 | Y |  |
| 59 | deoxyribonuclease i-like 3 | -1.784 | -1.881 | Y |  |
| 60 | dihydroxyacetone kinase 2 homolog (s. cerevisiae) | -1.643 | -1.377 | Y |  |
| 61 | elovl family member 7, elongation of long chain fatty acids (yeast) | 1.753 | 0.976 | Y |  |
| 62 | endothelin receptor type b | -1.454 | -1.55 | Y |  |
| 63 | enhancer of zeste homolog 2 (drosophila) | 1.694 | 1.405 | Y |  |
| 64 | epithelial cell transforming sequence 2 oncogene | 1.737 | 1.775 | Y |  |
| 65 | erbb receptor feedback inhibitor 1 | -1.512 | -1.372 | Y |  |
| 66 | extracellular matrix protein 1 | -1.503 | -2.059 | Y |  |
| 67 | fat tumor suppressor homolog 1 (drosophila) | 1.689 | 0.8 | Y |  |
| 68 | fatty acid binding protein 5 (psoriasis-associated) | 1.317 | 0.526 | Y |  |
| 69 | fc fragment of igg, low affinity iib, receptor (cd32) | -1.607 | -0.968 | Y |  |
| 70 | fibroblast growth factor 21 | 1.812 | 1.133 | Y |  |
| 71 | flavin containing monooxygenase 3 | -1.369 | -0.79 | Y |  |
| 72 | forkhead box q1 | -1.775 | 0.937 | N |  |
| 73 | gap junction protein, alpha 1, 43kda | -1.717 | 0.93 | N |  |
| 74 | glutamic-oxaloacetic transaminase 1, soluble (aspartate aminotransferase 1) | -1.7 | -1.625 | Y |  |
| 75 | glutaminase 2 (liver, mitochondrial) | -1.79 | -1.692 | Y |  |
| 76 | glutathione s-transferase mu 1 | 1.811 | -1.304 | N |  |
| 77 | glycine dehydrogenase (decarboxylating) | -1.759 | -1 | Y |  |
| 78 | glycoprotein m6a | -1.731 | -2.378 | Y |  |
| 79 | growth arrest-specific 2 like 3 | 1.654 | 1.569 | Y |  |
| 80 | hgf activator | -1.675 | -1.539 | Y |  |
| 81 | histidine ammonia-lyase | -1.813 | -1.223 | Y |  |
| 82 | holliday junction recognition protein | 1.531 | 1.623 | Y |  |
| 83 | hydroxyacid oxidase 2 (long chain) | 1.225 | -1.352 | N |  |
| 84 | hydroxysteroid (11-beta) dehydrogenase 1 | -1.702 | -1.09 | Y |  |
| 85 | hydroxysteroid (17-beta) dehydrogenase 6 homolog (mouse) | -1.707 | -1.11 | Y |  |
| 86 | immunoglobulin j polypeptide, linker protein for immunoglobulin alpha and mu polypeptides | -1.475 | -1.817 | Y |  |
| 87 | inhibitor of dna binding 1, dominant negative helix-loop-helix protein | -1.57 | -1.232 | Y |  |
| 88 | insulin-like growth factor 1 (somatomedin c) | -1.759 | -1.358 | Y |  |
| 89 | insulin-like growth factor binding protein 2, 36kda | -1.784 | -1.337 | Y |  |
| 90 | insulin-like growth factor binding protein 3 | -1.74 | -1.668 | Y |  |
| 91 | kinesin family member 20a | 1.762 | 1.551 | Y |  |
| 92 | kruppel-like factor 10 | -1.554 | -1.701 | Y |  |
| 93 | kynureninase (l-kynurenine hydrolase) | -1.768 | -1.923 | Y |  |
| 94 | kynurenine 3-monooxygenase (kynurenine 3-hydroxylase) | -1.783 | -1.724 | Y |  |
| 95 | lactate dehydrogenase d | 1.544 | -1.233 | N |  |
| 96 | leucine-rich repeat-containing g protein-coupled receptor 5 | 1.816 | 0.8 | Y |  |
| 97 | leukocyte cell-derived chemotaxin 2 | 1.794 | -0.873 | N |  |
| 98 | macrophage receptor with collagenous structure | -0.966 | -2.478 | Y |  |
| 99 | mad2 mitotic arrest deficient-like 1 (yeast) | 1.757 | 1.22 | Y |  |
| 100 | metallothionein 1e | -1.565 | -1.366 | Y |  |
| 101 | metallothionein 1f | -1.593 | -1.697 | Y |  |
| 102 | monoacylglycerol o-acyltransferase 2 | 1.475 | -1.627 | N |  |
| 103 | n-acetyltransferase 8 (gcn5-related, putative) | 1.765 | -1.178 | N |  |
| 104 | n-myc downstream regulated 1 | 1.49 | 0.697 | Y |  |
| 105 | nad(p)h dehydrogenase, quinone 1 | 1.659 | 0.79 | Y |  |
| 106 | nicotinamide n-methyltransferase | -1.686 | -1.661 | Y |  |
| 107 | notum pectinacetylesterase homolog (drosophila) | 1.604 | 0.654 | Y |  |
| 108 | oncoprotein induced transcript 3 | -1.614 | -2.455 | Y |  |
| 109 | orosomucoid 1 | -1.774 | -1.066 | Y |  |
| 110 | parathyroid hormone 1 receptor | -1.445 | -2.469 | Y |  |
| 111 | pdz binding kinase | 1.698 | 1.475 | Y |  |
| 112 | pdzk1 interacting protein 1 | 1.79 | 0.693 | Y |  |
| 113 | peroxisomal membrane protein 2, 22kda | -1.625 | -1.168 | Y |  |
| 114 | phenazine biosynthesis-like protein domain containing | -1.726 | -1.184 | Y |  |
| 115 | phosphoenolpyruvate carboxykinase 1 (soluble) | -1.803 | -1.111 | Y |  |
| 116 | phosphoserine aminotransferase 1 | 1.333 | -2.017 | N |  |
| 117 | plasmalemma vesicle associated protein | -1.685 | 1.259 | N |  |
| 118 | polycystic kidney disease 2 (autosomal dominant) | -1.6 | -1.809 | Y |  |
| 119 | potassium intermediate/small conductance calcium-activated channel, subfamily n, member 2 | -1.562 | -2.132 | Y |  |
| 120 | prolyl 4-hydroxylase, alpha polypeptide ii | -1.575 | 1.224 | N |  |
| 121 | protein regulator of cytokinesis 1 | 1.746 | 1.64 | Y |  |
| 122 | protocadherin 24 | 1.563 | -1.9 | N |  |
| 123 | r-spondin 3 homolog (xenopus laevis) | -1.725 | -2.297 | Y |  |
| 124 | retinol dehydrogenase 16 (all-trans) | -0.054 | -1.592 | Y |  |
| 125 | ring finger protein 43 | 1.787 | 1.435 | Y |  |
| 126 | roundabout, axon guidance receptor, homolog 1 (drosophila) | 1.724 | 1.172 | Y |  |
| 127 | serglycin | -1.12 | -1.565 | Y |  |
| 128 | serine dehydratase | -1.799 | -1.833 | Y |  |
| 129 | serum deprivation response (phosphatidylserine binding protein) | -1.538 | -1.928 | Y |  |
| 130 | shc sh2-domain binding protein 1 | 1.578 | 1.24 | Y |  |
| 131 | shugoshin-like 2 (s. pombe) | 1.815 | 1.422 | Y |  |
| 132 | solute carrier family 10 (sodium/bile acid cotransporter family), member 1 | -1.515 | -1.103 | Y |  |
| 133 | solute carrier family 17 (sodium phosphate), member 2 | -1.725 | -1.145 | Y |  |
| 134 | solute carrier family 22 (organic anion transporter), member 7 | -1.656 | -0.969 | Y |  |
| 135 | solute carrier family 22 (organic cation transporter), member 1 | 1.782 | -1.353 | N |  |
| 136 | solute carrier family 37 (glucose-6-phosphate transporter), member 4 | -1.693 | -1.349 | Y |  |
| 137 | solute carrier family 44, member 3 | 1.715 | 0.665 | Y |  |
| 138 | sp5 transcription factor | 1.773 | 0.843 | Y |  |
| 139 | squalene epoxidase | 1.587 | 1.63 | Y |  |
| 140 | st6 beta-galactosamide alpha-2,6-sialyltranferase 1 | -1.177 | -1.664 | Y |  |
| 141 | t-cell immunoglobulin and mucin domain containing 4 | -0.802 | -2.549 | Y |  |
| 142 | tocopherol (alpha) transfer protein | -1.814 | -1.248 | Y |  |
| 143 | transferrin receptor 2 | -1.783 | -1.121 | Y |  |
| 144 | transmembrane protein 45b | 1.545 | 0.977 | Y |  |
| 145 | tubulin, beta 2a | 1.68 | 0.95 | Y |  |
| 146 | tyrosine aminotransferase | -1.478 | -1.611 | Y |  |
| 147 | ubiquitin-conjugating enzyme e2c | 1.728 | 1.476 | Y |  |
| 148 | ubiquitin-conjugating enzyme e2t (putative) | 1.612 | 1.955 | Y |  |
| 149 | urocanase domain containing 1 | -1.766 | -1.222 | Y |  |
| 150 | vanin 1 | 1.639 | -1.47 | N |  |
| 151 | vasoactive intestinal peptide receptor 1 | -1.768 | -1.962 | Y |  |
